# Earliest expansion of animal husbandry beyond the Mediterranean zone in the sixth millennium BC

**DOI:** 10.1038/s41598-017-07427-x

**Published:** 2017-08-02

**Authors:** Jonathan Ethier, Eszter Bánffy, Jasna Vuković, Krassimir Leshtakov, Krum Bacvarov, Mélanie Roffet-Salque, Richard P. Evershed, Maria Ivanova

**Affiliations:** 1 Institut für Ur- und Frühgeschichte und Vorderasiatische Archäologie, Universität Heidelberg, Marstallhof 4, 69117 Heidelberg, Germany; 2 Römisch-Germanische Kommission des Deutschen Archäologischen Instituts, Palmengartenstr. 10-12, 60325 Frankfurt, Germany; 3 Department of Archaeology, Faculty of Philosophy, University of Belgrade, Čika Ljubina 18-20, 11000 Belgrade, Serbia; 4 Department of Archaeology, Faculty of History, Sofia University St. Kliment Ohridski, 15 Tzar Osvoboditel Boulevard, 1504 Sofia, Bulgaria; 5 National Institute of Archaeology and Museum, Bulgarian Academy of Sciences, 2 Saborna St., 1000 Sofia, Bulgaria; 6 Organic Geochemistry Unit, School of Chemistry, University of Bristol, Cantock’s Close, Bristol, BS8 1TS United Kingdom

## Abstract

Since their domestication in the Mediterranean zone of Southwest Asia in the eighth millennium BC, sheep, goats, pigs and cattle have been remarkably successful in colonizing a broad variety of environments. The initial steps in this process can be traced back to the dispersal of farming groups into the interior of the Balkans in the early sixth millennium BC, who were the first to introduce Mediterranean livestock beyond its natural climatic range. Here, we combine analysis of biomolecular and isotopic compositions of lipids preserved in prehistoric pottery with faunal analyses of taxonomic composition from the earliest farming sites in southeast Europe to reconstruct this pivotal event in the early history of animal husbandry. We observe a marked divergence between the (sub)Mediterranean and temperate regions of Southeast Europe, and in particular a significant increase of dairying in the biochemical record coupled with a shift to cattle and wild fauna at most sites north of the Balkan mountain range. The findings strongly suggest that dairying was crucial for the expansion of the earliest farming system beyond its native bioclimatic zone.

## Introduction

The wild progenitors of the main domestic animals in the Old World are endemic to regions with Mediterranean climate and are adapted to withstand prolonged hot summer droughts and mild but wet winters^1, 2^. Since domestication, farmers have brought sheep, goats, pigs and cattle to an enormous variety of environments, from semi-deserts to sub-arctic regions. Their present-day distribution, pushed out to the boundaries of the world inhabitable by humans, was mediated through human protection and breeding of animals that thrive under conditions often not tolerated by their wild ancestors. The first steps in this process can be traced back to the farming pioneers of the Balkans who penetrated beyond the borders of the sub-Mediterranean zone of Europe in the early centuries of the sixth millennium BC. How the first Balkan herders succeeded in extending the habitat of their livestock is an intriguing question with pivotal importance for the early history of human-animal relationships.

During the later seventh and early sixth millennia BC permanent farming settlements, similar to contemporary sites in the core areas of domestication in southwest Asia, became established in the (sub-)Mediterranean southern Balkans^3, 4^. The spread of farming economy into the temperate northern parts of the peninsula, however, was accompanied by pronounced changes, including higher (probably seasonal) residential mobility, smaller community sizes and a loss of sophistication in architecture and material culture^5–8^, a phenomenon which has been designated in the archaeological literature as “the First Temperate Neolithic”. Although the adaptation of herding economy to new bioclimatic conditions has been recognised as a major component of this phenomenon^8–14^, the human strategies which promoted it have remained uncertain.

Since early farmers kept domestic animals primarily for food and were dependent on them for survival, we can assume that their use of animal foodstuffs was finely adjusted to the animals’ productivity under different ecological conditions. Differences in the use of meat and milk at different latitudes in the Balkans are therefore likely to reflect regional adaptations in the course of the northward expansion of herding. Herein, we combine organic residue analysis of ceramic vessels with faunal data on taxonomic composition to investigate variation in the use of animal products across the different bioclimatic zones of the Balkans.

## Results

Faunal remains are widely used in archaeology as a proxy for past interactions between people and animals^15–17^. Datasets with taxonomic frequencies are available from the majority of recently excavated early farming sites in Southeast Europe. These datasets are generally dominated by food species and provide information on the relative proportions of consumed animals, but do not directly reflect differences in the used products (milk, meat, and fat). Investigations of fat residues in archaeological pottery can directly verify the use of particular animal foodstuffs, and differentiate between dairy, ruminant carcass and non-ruminant carcass fats, as well as a range of other commodities, such as aquatic resources or beeswax^18–22^. Lipid residues and faunal remains are thus complementary strands of evidence for recovering past practices of animal exploitation^23^.

### Regional patterns in lipid residues

**Figure 1:**
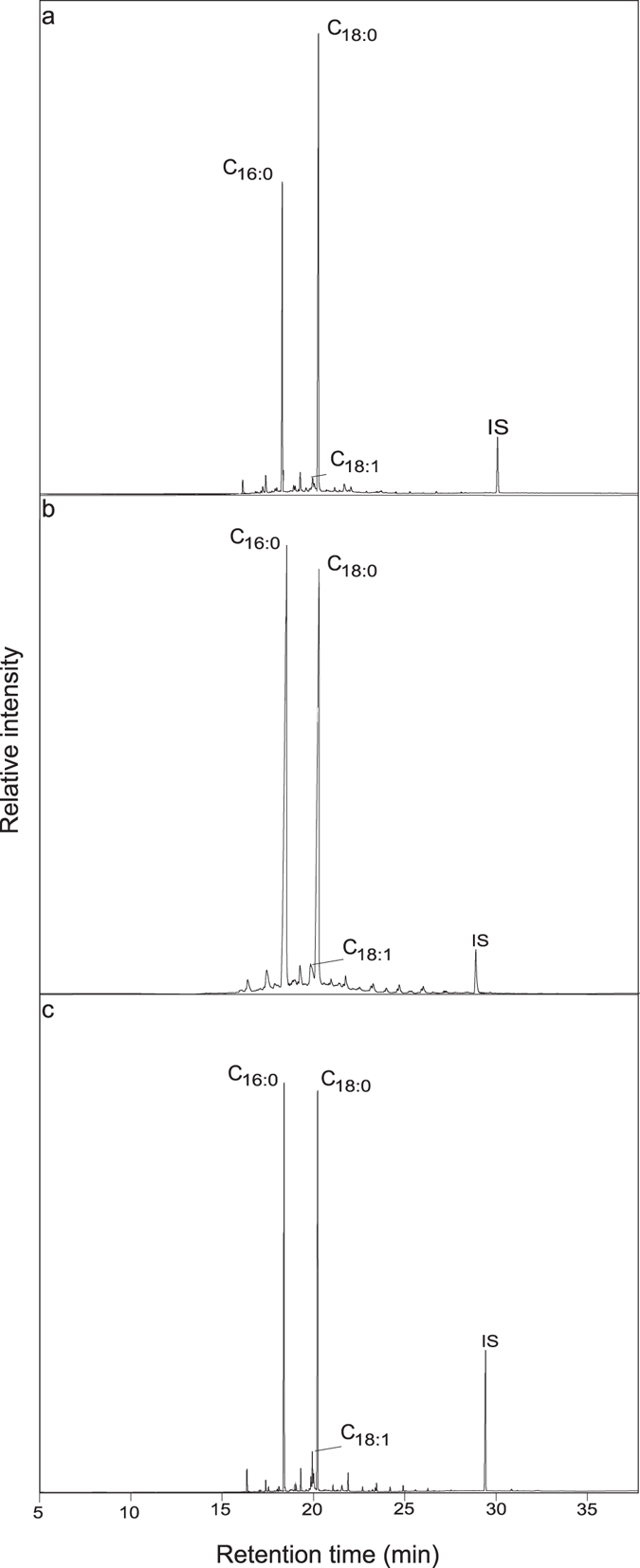
Gas chromatograms of TLEs from pottery from the three regions of study showing high concentration of palmitic acid (C_16:0_) and stearic acid (C_18:0_): (**a**) Ecsegfalva 23, sample 16, (**b**) Grivac, sample 17, and (**c**) Yabalkovo, sample 38. IS – internal standard: C_34_ *n*-tetratriacontane.

From the 290 pottery samples analysed in this study, 22% (n = 64) provided identifiable lipid residues with concentrations up to 5679 μg g^−1^ (mean 802 μg g^−1^, for the sherds containing lipids). This preservation rate is comparable with previous studies of prehistoric pottery from the Mediterranean^24^, but lower than the proportions of over 50% of sherds with extracted lipids from Central Europe^25^. All sites were nearly equally represented with ca. 20% to 35% of the samples containing identifiable lipids of archaeological origin, the only exception being Divostin, where only 7% of sherds contained observable lipids (Table 1). The lipid extracts were dominated by fatty acids derived from animal fat, i.e. palmitic acid (*n*-hexadecanoic acid; C_16:0_) and stearic acid (*n-*octadecanoic acid; C_18:0_; Fig. 1, Supplementary Table S1). Biomarkers characteristic of plant material were not detected.

**Table 1:** Sample information and summary of organic residue analysis of pottery

Sites	Dating	Potsherds analysed	Residues Detected		Non-ruminant adipose fats		Ruminant adipose fats		Ruminant dairy fats	
			n	%	n	%	n	%	n	%
**C. Hungarian plain**										
Alsonyek	5800-5730 BC	43	14	32	—	—	5	36	9	**64**
Ecsegfalva 23	5800-5650 BC	41	8	20	1	12	2	25	5	**63**
**B. Northern Balkans**										
Blagotin	6200-6000 BC	43	9	21	—	—	2	22	7	**78**
Grivac	6200-6000 BC	33	6	18	—	—	3	50	3	**50**
Divostin	6000-5700 BC	43	3	7	2	67	0	0	1	**33**
**A. Southern Balkans**										
Yabalkovo	6000-5700 BC	42	15	35	3	20	9	60	4	**20**
Nova Nadezhda	6000-5600 BC	45	9	20	1	11	8	89	—	—
Total		290	64	22	7	—	30	—	28	—

(descriptions of sites, radiocarbon dates, stratigraphic contexts, and sample descriptions are given in the Supplementary Information).

The identification of the various commodities was based on comparison of the carbon isotope compositions of archaeological fatty acids to those of modern reference animal fats. To eliminate differences in diets between the modern reference and the prehistoric animals caused by environmental and dietary factors, the Δ^13^C values (δ^13^C_18:0_ − δ^13^C_16:0_) of the archaeological samples were calculated and compared to that of modern reference fats^25–29^. All archaeological lipids derived from ruminant or non-ruminant animals. Potential contributors of non-ruminant fats include domestic pig, wild boar, fowl and domestic dog. No biomarkers of aquatic resources, such as ω-(*o*-alkylphenyl)alkanoic acids, were detected.

**Figure 2:**
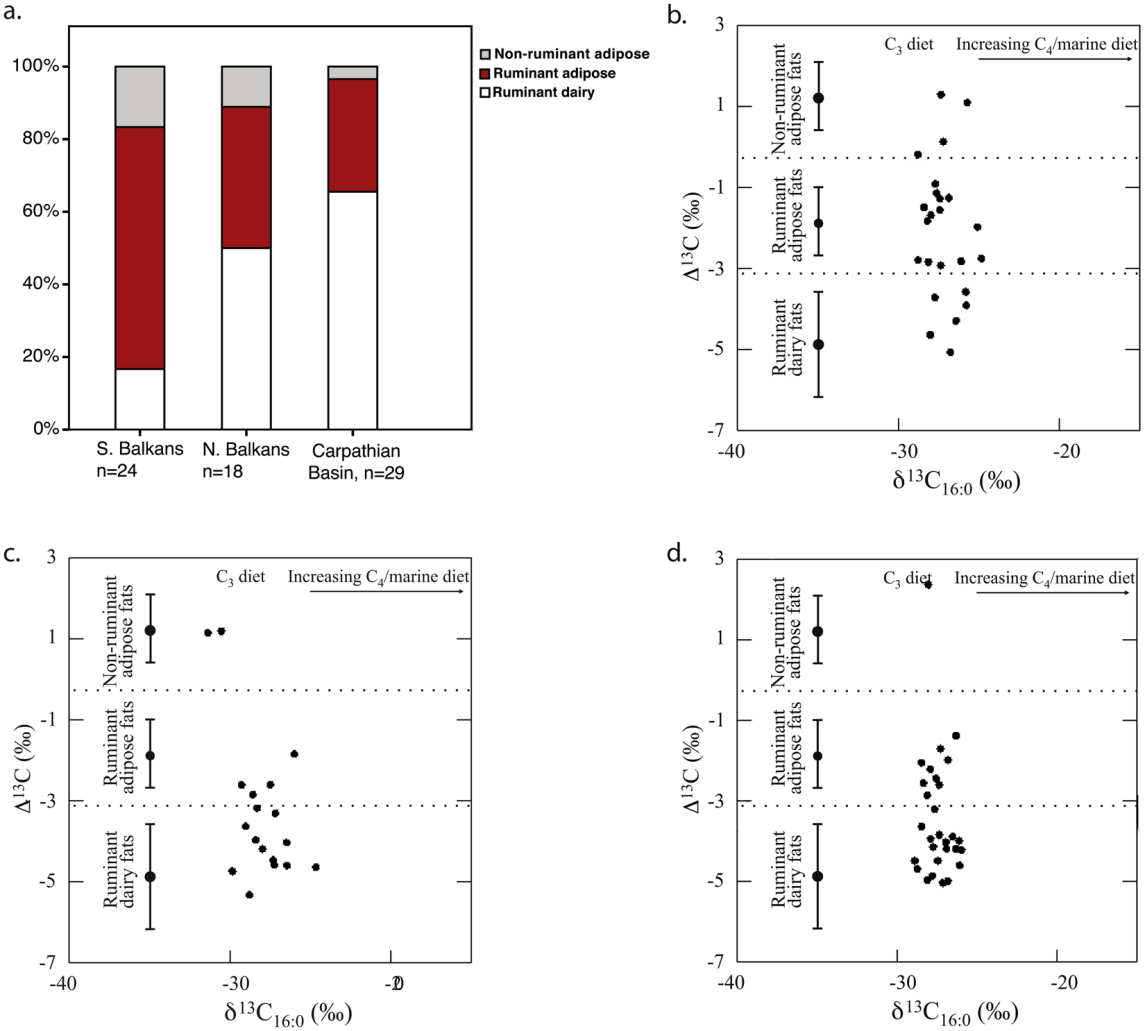
Regional differences in Δ^13^C values ( = δ^13^C_18:0_ − δ^13^C_16:0_) of archaeological lipid residues in pottery. (**a**) proportions of lipid residues according to commodity type; (**b**) Δ^13^C values of archaeological lipid residues from sites in (b) the southern Balkans, (**c**) northern Balkans (**d**) the Carpathian basin (including published data for Ecsegfalva^32^). Each data point represents an individual vessel.

The difference between the southern and northern part of the Balkans in respect to the distribution of commodities among the lipid residues is highly significant (p = .002, Fisher’s exact test). Over 70% (n = 17) of all animal fat residues detected in pottery from the southern part of the Balkans in the present study plot within the range of ruminant adipose fats (Fig. 2b). At Yabalkovo, all three sources of animal fat were identified, although non-ruminant and dairy residues were represented only in low proportions (20% each). The pattern is even more pronounced in the distribution of residues from Nova Nadezhda, where dairy fats were not detected and the proportion of ruminant adipose fats reached 89%. These findings agree well with previously published data from early farming sites in other areas with Mediterranean climate, e.g. the Levant, Syria, Anatolia, Greece and Italy^24, 27, 30, 31^. We can denote this type of early livestock use, reflected in strong predominance of ruminant carcass fat and low incidence or absence of dairy fat among the residues in ceramic vessels, the “Mediterranean type”.

The lipid residues from sites in the northern part of the Balkans and in the Hungarian Plain, characterized by transitional and sub-continental climate, demonstrate a different pattern (Fig. 2c,d). Notable similarities exist between distant sites in present-day Serbia and Hungary (with the only exception of Divostin, which is not representative because of the very small number of sherds with residues, n = 3). Residues classified as non-ruminant fats are nearly absent, which agrees with the very low percentages of pigs in the faunal assemblages. With 60% (n = 24), dairy fats are by far the most abundant class of animal fat residues. The results obtained in the current study represent the first substantial dataset from the earliest farming sites in the temperate zone. They are comparable to the results of a previous study of pottery from Ecsegfalva 23 with n = 7 sherds with residues^32^. The type of animal product exploitation reflected in a residue record with a strong emphasis on dairy fats and nearly absent non-ruminant residues can be provisionally assigned as the “Temperate type”.

### Faunal data on species preferences

**Figure 3:**
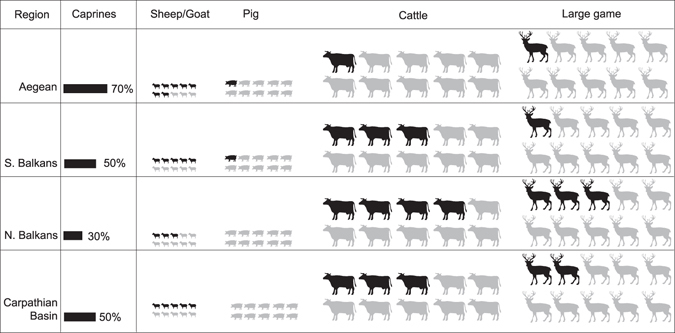
General trends in the exploitation of the most abundant mammalian taxa. Faunal data from early farming sites in the Aegean, the Balkans and the Carpathian basin, symbol size corresponds to relative body size of species, each symbol represents 10% of NISP. See Supplementary Table S2 for the complete dataset.

The taxonomic compositions of the faunal assemblages from early farming sites in the interior of the southern Balkans are similar to these from the Mediterranean littoral of the peninsula, with a somewhat lower proportion of caprines and more cattle compared to the Aegean (Fig. 3). The faunal data from the northern Balkans, in contrast, suggests a clearly different strategy of animal foodstuff supply. The proportion of caprines decreased compared to the south and cattle assumed a major role among livestock in the northern Balkans, clearly predominating as a supplier of meat when their body mass is considered. Together with the increasing importance of large game, this change in taxonomic frequencies highlights a shift to ‘‘local’’ food species (animals which are native to or have close endemic relatives in temperate Europe). Surprising is the nearly complete absence of domestic pigs (represented by only a few percent of NISP), since pigs must have thrived in the temperate forests and marshlands. One possible explanation for the scarcity of pigs in the northern Balkans is seasonal mobility of herds: although pig transhumance is known from the ethnographic record^33^, pigs are in general untypical for the seasonally relocated herds. It has been previously argued that the preference for mobile fauna in the northern Balkans relates to other mobility indicators, such as smaller community size, lower settlement permanence, less substantial or semi-subterranean dwellings, and possibly decreasing reliance on plant cultivation^34^. Seasonal mobility is also tentatively suggested by the age distribution of caprines and by the absence of migratory species among birds in the faunal assemblage from Blagotin, indicating cold season (late autumn to late spring) presence at this location^35^. However, other causes for the decline in pig husbandry also need to be considered.

**Figure 4:**
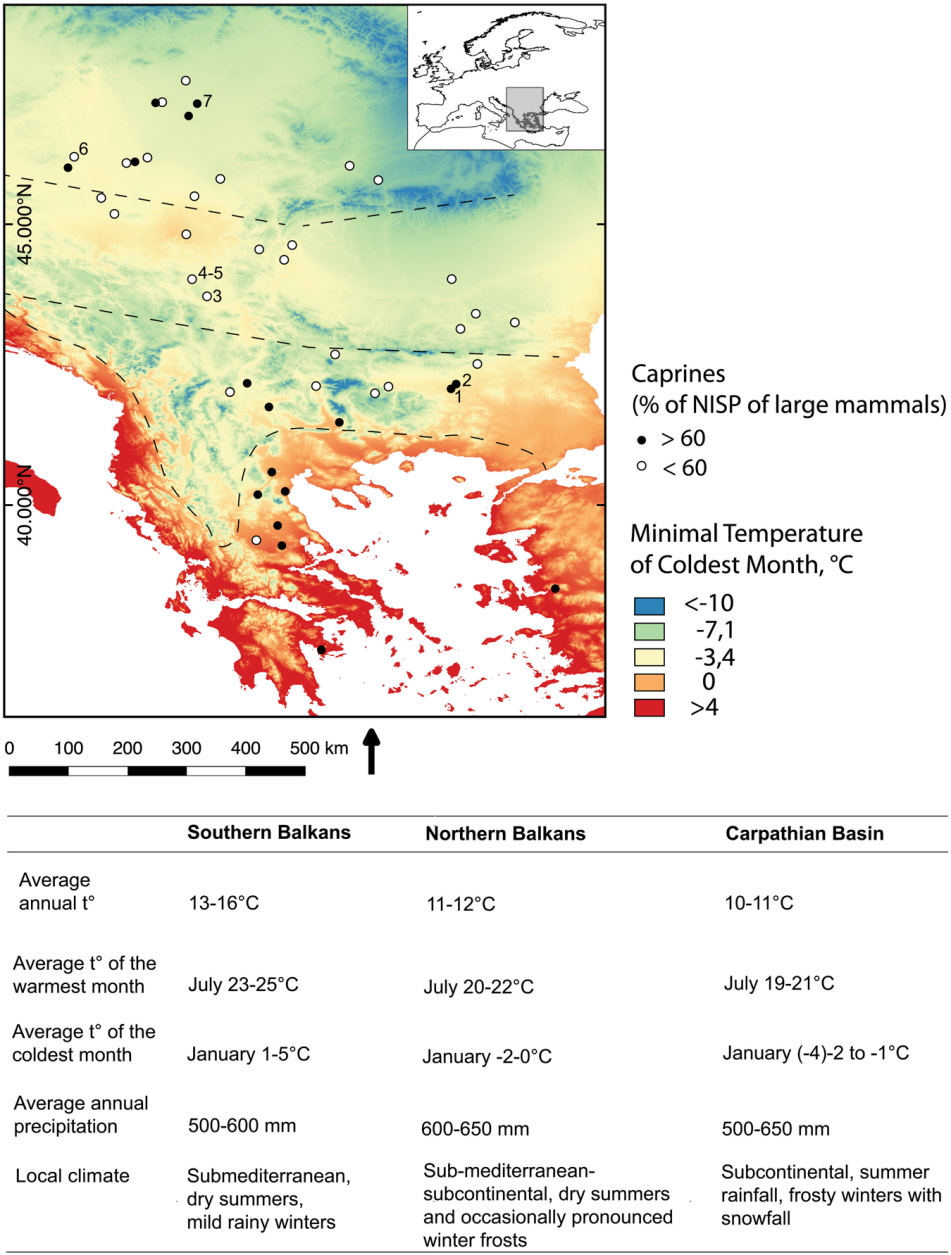
Summary of climatic conditions in Southeast Europe. Dots indicate the location of faunal assemblages included in this study (see Supplementary Table S2); black dots – sites where caprines were represented with >60% of NISP of the large- and middle-sized domestic and wild mammalian taxa, white dots – caprines <60%. The dotted lines indicate regional boundaries between the Aegean/Adriatic littoral, southern Balkans, northern Balkans and Carpathian Basin. Numbers show location of sites where organic residue analyses were performed 1. Yabalkovo, 2. Nova Nadezhda, 3. Blagotin, 4. Divostin, 5. Grivac, 6. Alsónyék, 7. Ecsegfalva 23. (Climate data from: Hijmans, R. J., S. E. Cameron, J. L. Parra, P. G. Jones and A. Jarvis. 2005. Very high resolution interpolated climate surfaces for global land areas. International Journal of Climatology 25(15): 1965-1978; http://worldclim.org/, Layer BIO6 = Min Temperature of Coldest Month, interpolations of observed data, representative of 1960–1990). The map was created using QGIS Version 2.8.9-Wien (http://qgis.org/de/site/).

Similar taxonomic abundances are characteristic of the faunal distributions from the Hungarian Plain. The small ruminants seem more numerous here compared to the northern Balkans, though this picture is biased by several sites in the northern periphery of the region which focused intensively on caprines (70 to 80% of NISP; Fig. 4). Since the swampy wetland environments of the Danube and Tisza plains are adverse to sheep husbandry, these assemblages have often been described as puzzling^36, 37^ and the preference for caprines has been attributed to emotional motives of the early farmers, who adhered to the pastoral traditions of their Mediterranean ancestors against all odds^38^. However, a large area between the Aegean and the Carpathian basin where ‘‘local’’ species (cattle and large game) prevailed in the faunal remains indicates that continuity of herding practices is not a completely satisfactory explanation for the sites with high proportions of caprines in eastern Hungary (Fig. 4). Rather, the latter can be considered more anomalous than typical for the temperate zone. A likely pragmatic reason for the prevalence of caprines at some sites in Hungary, despite the unfavourable environment, are problems with livestock overwintering due to snow cover and shorter plant growing season. “Caprine” sites are situated in grassland areas, where large game hunting was not as rewarding as on the forest margins^39^. In view of their higher reproductive rate, opting for caprines (rather than cattle) may be an advantage when herders are forced to slaughter many of their animals before or during winter because of limited fodder, since the annual growth rate in number of heads in sheep herds and goat herds is higher than that of cattle^40^. Moreover, in competition with pigs, caprines possess an advantage which becomes crucial under conditions of decreasing animal productivity – milk exploitation yields up to four times higher yield of food energy compared to hunting or growing animals for meat^41^.

## Discussion

The interior of the Balkan Peninsula and the adjacent Hungarian Plain occupy an intermediate position between the Mediterranean and the Central European bioclimatic regions. The present-day distribution of thermophilous mixed deciduous broadleaved forests indicates fluid transitions of temperature and precipitation, from mild winter temperatures in the south and occasional winter frosts in the northern parts of the peninsula to regular periods of winter frost and snow in the Hungarian plain^42^. Reconstructions of warmest month, coldest month and mean annual surface air temperatures, based on fossil pollen data, suggest that winter temperatures in southeast Europe have generally remained at the same level from 6000 calBC through to the present (Fig. 4)^43^.

Farmers and their domestic animals spread fast, in c. 10 human generations, across these biogeographic zones from sub-Mediterranean Macedonia to the northern limits of the temperate Carpathian Basin^10, 44–46^. The increasingly unfavourable climate, and particularly the long frosty winters, had certainly an impact on Mediterranean livestock. Climate affects animals in a direct way through thermoregulation, and indirectly, through trophic links (e.g. pasture quality and biomass)^47^. Climate-driven selective pressure brings forward changes in the genome, such as the adaptation to feed scarcity through accumulation of fat as energy reserve, or through a seasonal decline in appetite and the ability to reduce metabolism in winter^48, 49^. Genetically-determined improvements of feed efficiency, pigmentation, coat and body size are further common adaptations to harsh environments.

However, since genetic changes do not occur immediately, farmers spreading into higher latitudes in the Balkans are likely to have initially experienced difficulties in maintaining the productivity of all domesticates, and in particular of sheep, which do not have native wild relatives in Europe and, being grazers, are not adapted to feed in the forested environments of the Balkan interior. The most serious hurdle to overcome was the provision of feed under conditions of a shorter period of plant growth and deep snow impeding access to grass for weeks in winter. Spring temperatures have also been recognized for their effect on animal productivity. For example, 1 °C decrease in mean spring temperature has been estimated to reduce lamb autumn body mass by nearly 0.4 kg^50^. One further climate-related parameter, directly associated with animal productivity, is the seasonality of mating and birth, which is determined by day-length through a genetically controlled mechanism for sheep and through feed availability for cattle^51, 52^.

Before genetically adapted breeds became established, these obstacles must have been overcome by techniques of herd management, such as control of mating, strategic culling, adjustments in the species composition, interbreeding with wild populations, and labour-intensive feeding practices (stalling, foddering, change of pastures). Equally important must have been, however, adjustments in the use of animal foodstuffs and in the techniques of food processing. For example dairying, a practice familiar to the early farmers of southwest Asia and the Mediterranean^24, 27, 53^, is highly efficient in increasing the yields of animal husbandry. Milk pastoralism can provide up to four times higher yields of food energy in comparison to growing animals for meat^41^. T. Ingold lucidly explains the higher productivity of milking in comparison to meat exploitation by the respective position of the herders in the food chain. Pastoralist who milk their animals intercept the chain of conversions from grass to milk, and from milk to meat, at an earlier point, thus avoiding the net loss of energy that takes place at the latter stage of this conversion^54^.

Our investigation of animal lipids preserved in pottery and of taxonomic frequencies of large- and medium-sized mammalian species shows differences in the use of animal products between the Aegean and the southern Balkans, on the one hand, and the northern Balkans and the Hungarian Plain on the other hand. We observe a significant increase of dairy fats in the biochemical record coupled with a decline in caprines at most sites north of the Balkan mountain range. Our study indicates that dairying, being a highly efficient way to counterbalance reduced productivity of animal husbandry, was instrumental to the spread of the Mediterranean livestock system into the temperate areas of the Balkans, and from there across the entire European continent. The general distinction of a “Mediterranean” and a “Temperate” type of early animal husbandry outlined in the present study does not rule out the existence of small**-**scale regional variation. Previous studies of lipids in early ceramics in the Mediterranean, for example, suggest that a high proportion of dairy residues might be characteristic for early farmers’ habitations in caves and rock-shelters^24, 55, 56^, possibly pointing at the presence of specialized seasonal herding sites.

Finally, dairying represents not only an efficient economic strategy, but also provides an important dietary component. Milk was one of the main potential sources of dietary fat for the first Balkan farmers, together with lard, oily fish and probably waterfowl. Having in mind that pigs were barely represented in the faunal assemblages from the northern Balkans and the Carpathian Basin, and that non-ruminant lipids were very rare among the residues in pottery, we can regard dairying as a key strategy of obtaining fat in this geographic region. Fresh milk in small-scale non-industrial animal husbandry is a seasonal product. Peak lactation in temperate climates generally coincides with the warm season, when pastures can support the high nutritional requirements of lactating stock^51^. Being a perfect medium for pathogenic microbial growth, in the hot months milk spoils to an ill-smelling liquid within hours, if not consumed or heated and processed in a controlled manner (for example through controlled souring by lactic bacteria). The low frequency or absence of the −13,910*T allele, associated with continued production of lactase into adulthood (and thus with the ability to digest fresh milk), in hunter-gatherer and early farming populations in Europe^57–61^, suggests that high-lactose fresh milk could not have been consumed in large quantities by the first farmers. Hence, most of the milk must have been processed without delay both to reduce its lactose content and to convert it into a storable year-round staple food. The increase in dairying observed in this study thus represents a remarkable example of complex cultural behavior employed to circumvent the limitations of environment and of human and animal biology.

## Methods

### Organic residue analysis

#### Sampling

In this study, we analysed a total of 290 potsherds from seven pottery assemblages dating to c. 6100–5700 calBC (Table 1). The sherds derive from well-documented and published excavations in Bulgaria (Yabalkovo and Nova Nadezhda), Serbia (Blagotin, Divostin and Grivac), and Hungary (Alsónyék and Ecsegfalva 23), which have provided the earliest Neolithic assemblages in their respective geographic area^35, 62–67^ (Supplementary materials). Sherds were selected following two main criteria – an undisturbed stratigraphic context and a representative spectrum of wares and shapes (including diverse types of plates, bowls and jars); they were mostly diagnostic rim fragments (73%).

#### Lipid extraction and derivatisation

Lipid extractions were performed using direct acidified methanol extraction, according to the protocol of Correa-Ascencio and Evershed^68^. Using a modelling drill, a small area of each fragment was cleaned to remove modern contaminants (from soil burial, excavation and post-excavation handling) and a sample of ca. 2 g was taken. The sherd fragment was crushed into a fine powder, weighed and an internal standard of *n*-tetratriacontane (20 μl, 1.0 mg ml^−1^ solution) was added. Lipids were extracted from the powder by adding 5 ml of a H_2_SO_4_-MeOH (4% sulphuric acid/methanol) solution (δ^13^C known), heated for 1 h at 70 °C, whirl mixed every 10 min to ensure contact between solvent and powdered sherd. In order to help observe the solvent layers, the H_2_SO_4_-MeOH was transferred to a clean culture tube, where 2 ml of DCM extracted-double-distilled water was added. To ensure the recovery of lipids not fully solubilized by the methanol solution, hexane (2 ml portions) washing was performed 2x for the powdered sherd and 3x for the diluted methanol extract. All hexane extracts were combined and evaporated to dryness under a gentle stream of nitrogen. Prior to instrumental analysis one fourth aliquots of total lipid extracts (TLEs) diluted in hexane were blown down to dryness with nitrogen and derivatised with 20 μl of *N,O*-bis(trimethylsilyl)trifluoroacetamide (BSTFA containing 1% trimethylsilyl chloride) for 1 h at 70 °C to trimethylsilylate free alcohols.

#### Gas chromatography (GC), GC/mass spectrometry (GC/MS) and GC-combustion-isotope ratio MS (GC-C-IRMS)

Derivatised TLEs were blown to dryness then diluted with hexane and screened by GC to make preliminary identifications of components and quantify lipid concentrations in potsherds by reference to the *n*-tetratriacontane internal standard. Two different GC systems were used, employing either manual or autosampling injections: (1) Agilent Technologies 7820 series 2 with a silica capillary column (50 m × 0.32 mm) coated with a dimethyl polysiloxane stationary phase (J&W Scientific; CP-Sil 5 CB, 0.12 μm film thickness) or (2) Agilent 7890a with a capillary column (15 m × 0.32 mm) coated with a dimethyl polysiloxane stationary phase (J&W Scientific; DB1-HT, 0.1 μm film thickness). On both systems, the carrier gas was helium and a flame ionization detector (FID) was used to monitor the column effluent. The temperature programs began with an initial hold at 50 °C for 2 min followed by temperature programming at 10 °C min^−1^ and a final hold for 10 min at 350 °C (Agilent 7890a) or 300 °C (Agilent 7820). Collection of the data and quantification were performed using a Agilent Chemstation software.

Where structures of components were not identifiable based on GC alone, GC/MS analyses were performed on a ThermoFinnigan Trace MS quadrupole mass spectrometer coupled to a Trace GC. Using either manual or auto-injections samples were injected via the splitless mode onto a fused-silica capillary column (60 m × 0.32 mm) coated with a polymethylpolysiloxane stationary phase (Phenomenex; ZB1, 0.1 μm film thickness). The temperature programs for the GC began with an initial temperature held at 50 °C for 2 min and followed by a constant increase at a rate of 10 °C min^−1^ to 300 °C then held for 10 min. The MS was operated in electron ionisation (EI) mode (70 eV) with a GC interface temperature of 300 °C and a source temperature of 200 °C. The emission current was 150 μA and the data collection was between *m/z* 50–650 at 1.3 scans s^−1^. Xcalibur v3.0 software and a NIST spectra library were used to acquire, analyse and identify separated compounds. The same samples were screened for ω-(*o*- alkylphenyl)alkanoic acids using GC/MS selected ion monitoring (SIM; *m/z* 105, 262, 290, 318 and 346). The extracts were injected in the same GC as above equipped with a fused-silica capillary column (60 m × 0.32 mm) coated with a 50% cyanopropyl-methylpolysiloxane stationary phase (Varian, Factor Four; VF-23 ms, 0.15 μm film thickness). The GC oven temperature was programmed from 50 °C, following an isothermal hold for 2 min, to 100 °C at 10 °C min^−1^, and then to 240 °C at 4 °C min^−1^, following by an isothermal hold at 240 °C for 15 min. The extracts dominated by C_16:0_ and C_18:0_ fatty acids and free from plant lipids (e.g. long-chain *n*-alkanols and long-chain *n*-alkanoic acids), were analysed in duplicate by GC-C-IRMS to obtain their individual δ^13^C values. GC-C-IRMS analyses used an Agilent 6890 GC coupled to a Finnegan MAT Delta^plus^ XL mass spectrometer via a Finnigan MAT GCCIII interface. Samples were injected using a CTC A200S autosampler in splitless mode onto a Varian silica capillary column (CP-Sil 5 CB, 100% dimethylpolysiloxane with 0.12 μm film thickness, 50 m × 0.32 mm). The temperature program for the GC began with an initial temperature held at 50 °C for 2 min, followed by a constant increase in oven temperature at10 °C min^−1^ to 250 °C, followed by a 15 min hold.

## Faunal analyses

Taxonomic abundances in 39 early farming faunal assemblages from the Aegean, the Balkans and the Carpathian Basin were calculated based on NISP (number of identifiable specimens, identified to species level, with the exception of indistinguishable sheep/goat remains that represent the caprine subfamily; Supplementary Table S2). To limit bias through taphonomic history, recovery techniques and laboratory procedures, only the most ubiquitous large- and medium-sized mammalian taxa were considered. Because small assemblages of less than 500 NISP tend not to represent reliably the relative importance of animals^38, 39^, the assemblages included in this study exceed 890 NISP (with the exceptions of Nea Nikomedia n = 450 and Alsónyék n = 428).

Animal bones from excavations represent butchering and cooking remains and are thus directly related to the consumption of animal products. However, straightforward estimates of dietary contribution from taxonomic abundances based on NISP are not possible, since NISP neither corresponds to meat quantity, nor to the use of animals for meat and milk products. Other proxies, such as age-at-death (AtD) data, are available only for a handful of sites (Prodromos, Blagotin, Ecsegfalva 23, Miercurea Sibiului-Petriş, Foeni Salaș and Foeni Gaz)^37, 69–71^. The AtD data were considered in the interpretation of the organic residues in pottery; they are, however, insufficient for inter-regional comparisons. Although no calculation of dietary contributions was attempted in this study, we take into consideration the relative contributions of large- and medium-sized livestock and large game in the discussion of their importance in diet. We adopted a modified version of Clason’s “live-weight factor” approach to express the live weight of taxa in terms of sheep equivalents (sheep = 1, cattle = 28, pig = 1.2)^72, 73^. For large game, we use a factor of 22, based on the average of the aurochs (36), equid (24) and cervid (7) factors. These factors are only rough approximations of the relative meat contribution of taxa and do not represent actual body sizes or meat yields.

## Electronic supplementary material

Supplementary Information

## References

[1] Vigne JD (2011). The origins of animal domestication and husbandry: A major change in the history of humanity and the biosphere. Comptes Rendus Biologies.

[2] Zeder MA (2008). Domestication and early agriculture in the Mediterranean Basin: origins, diffusion, and impact. PNAS.

[3] Perles, C. The Early Neolithic in Greece. The First Farming Communities in Europe (Cambridge University Press, 2001).

[4] Nikolov, V. In *Neolithic of Southeastern Europe and its Near Eastern connections (Varia Archaeologica Hungarica 2*) (eds S. Bökönyi & P. Raczky) 191–200 (Institute of Archaeology of the Hungarian Academy of Sciences, 1989).

[5] Nandris, J. In *Problems in economic and social archaeology* (eds G. Sieveking, I. H. Longworth, & K. Wilson) 549–556 (Duckworth, 1976).

[6] Whittle, A. W. R. *Europe in the Neolithic: the creation of new worlds* (Cambridge University Press, 1996).

[7] Tringham, R. In *Europe’s* First Farmers (eds T. D. Price) 19–56 (Cambridge University Press, 2000).

[8] Nandris, J. In A Short Walk through the Balkans: The First Farmers of the Carpathian Basin and Adjacent Regions (eds M. Spataro & P. Biagi) 11–23 (2007).

[9] Halstead, P. In *The beginnings of agriculture* (eds A. Milles, D. Williams, & N. Gardner) 23–53 (British Archaeological Reports, 1989).

[10] Krauß, R., Marinova, E., Brue, H. D. & Weninger, B. The rapid spread of early farming from the Aegean into the Balkans via the Sub-Mediterranean-Aegean Vegetation Zone. *Quaternary International*, doi:10.1016/j.quaint.2017.01.019 (2017).

[11] Manning, K. *et al*. In *The origins and spread of domestic animals in Southwest Asia and Europe* (eds S. Colledge *et al*.) 237–252 (Left Coast, 2013).

[12] McClure SB (2013). Domesticated animals and biodiversity: early agriculture at the gates of Europe and long-term ecological consequences. Anthropocene.

[13] McClure SB (2015). The pastoral effect: niche construction, domesticated animals and the spread of farming in Europe. Current Anthropology.

[14] Orton D (2012). Herding, settlement, and chronology in the Balkan Neolithic. European Journal of Archaeology.

[15] Arbuckle BS (2014). Data sharing reveals complexity in the westward spread of domestic animals across Neolithic Turkey. PloS one.

[16] Colledge, S., Conolly, J., Dobney, K., Manning, K. & Shennan, S. *The origins and spread of domestic animals in Southwest Asia and Europe* (Left Coast Press, 2013).

[17] Russell, N. *Social zooarchaeology*. Humans and animals in prehistory (Cambridge University Press, 2012).

[18] Evershed RP (2008). Organic residue analysis in archaeology: the archaeological biomarker revolution. Archaeometry.

[19] Dudd SN, Evershed RP (1998). Direct demonstration of milk as an element of archaeological economies. Science.

[20] Evershed RP (1999). Lipids as carriers of anthropogenic signals from prehistory. Philosophical Transactions of the Royal Society.

[21] Evershed RP (2001). Chemistry of archaeological animal fats. Accounts of Chemical Research.

[22] Evershed, R. P. In *Orga*nic Mass *S*pectr*ometry in Art and Archaeology* (eds M. Perla Columnbini & F. Modugono) 391–432 (Chichester, 2009).

[23] Roffet-Salque, M. *et al*. From the inside out: Upscaling organic residue analyses of archaeological ceramics. *Journal of Archaeological Science: Reports* (2016).

[24] Debono Spiteri, C. *et al*. Regional asynchronicity in dairy production and processing in early farming communities of the northern Mediterranean. *PNAS* 13594–13599 (2016). 10.1073/pnas.1607810113 PMC5137723 27849595

[25] Salque M (2013). Earliest evidence for cheese making in the sixth millennium B.C. in northern Europe. Nature.

[26] Copley MS (2005). Dairying in Antiquity: III - Evidence from absorbed lipid residues dating to the British Neolithic. Journal of Archaeological Science.

[27] Evershed RP (2008). Earliest date for milk use in the Near East and southeastern Europe linked to cattle herding. Nature.

[28] Mukherjee, A. J., Copley, M. S., Berstan, R., Clark, K. A. & Evershed, R. P. In *The Zooarchaeology of Fats, Oils, Milk and Dairying - 9th ICAZ Conference* (eds J. Mulville & A. K. Outram) 77–93 (Oxbow Books, 2005).

[29] Dunne J (2012). First dairying in green Saharan Africa in the fifth millennium BC. Nature.

[30] Nieuwenhuyse O, Roffet-Salque M, Evershed R, Akkermans PM, Russell A (2015). Tracing pottery use and the emergence of secondary product exploitation through lipid residue analysis at Late Neolithic Tell Sabi Abyad (Syria). Journal of Archaeological Science.

[31] Salque M (2012). New insights into the Early Neolithic economy and management of animals in Southern and Central Europe revealed using lipid residue analyses of pottery vessels. Anthropozoologica.

[32] Craig OE (2005). Did the first farmers of central and eastern Europe produce dairy foods?. Antiquity.

[33] Albarella, U., Manconi, F. & Trentacoste, A. In *EthnoZooArchaeology: The Present Past of* Human-Animal *Relationships* (ed U. Albarella) 143–159 (Oxbow, 2011).

[34] Greenfield, H. J. & Jongsma, T. L. In Li*vin*g Well *To*gethe*r? Settlement and Materiality in the Neolithic of South-East and Central Europe* (eds D. W. Bailey, A. Whittle, & D. Hofmann) 108–130 (Oxbow Books, 2008).

[35] Greenfield HJ, Greenfield TLJ, Jezik S (2014). Subsistence and settlement in the Early Neolithic of temperate SE Europe: a view from Blagotin, Serbia. Archaeologia Bulgarica.

[36] Bartosiewicz, L. In Current Research in Animal Palaeopathology: Proceedings of the Second Animal Palaeopathology Working Group Conference. BAR International Series S1844 (eds Z. Miklíková & R. Thomas) 3–13 (Archaeopress, 2008).

[37] Bartosiewicz, L. In The Early Neolithic on the Great Hungarian Plain: investigations of the Körös culture site of Ecsegfalva 23, Co. Bekes. (*=Varia Archaeologica Hungarica 21*) (ed A. Whittle) 287–325 (2007).

[38] Bartosiewicz, L. In The First Neolithic Sites in Central/South-East European Transect. Volume III. The Körös Culture in Eastern Hungary. British Archaeological Reports, IBAR International Series 2334 (eds A. Anders & Z. Siklósi) 195-204 (Archaeopress, 2012).

[39] Bartosiewicz, L. In (Un)*settling the Neolithic* (eds D. Bailey & A. Whittle) 51–63 (Oxbow Books, 2005).

[40] Dahl, G. & Hjort, A. *Having Herds: Pastoral Herd Growth and Household Economy. Stockholm Studies in Social Anthropology No. 2* (University of Stockholm, 1976).

[41] Legge, A. J. In The Zooarchaeology of Fats, Oils, Milk and Dairying (Proceedings of the 9th ICAZ conference) (eds J. Mulville & A. Outram) 8–13 (Oxbow, 2005).

[42] Bohn, U. & Neuhäusl, R. Karte der natürlichen Vegetation Europas/Map of the Natural Vegetation of Europe. Maßstab/Scale 1: 2 500 000. (Landwirtschaftsverlag, 2014).

[43] Davis, B. A. S., Brewer, S., Stevenson, A. C. & Guiot, J. The temperature of Europe during the Holocene reconstructed from pollen data. *Quaternary Science Reviews* 1701–1716 (2003).

[44] Domboróczki, L. In Die Neolithisierung Mitteleuropas (The spread of the Neolithic to Central Europe). Internationale Tagung Mainz, 24–26. Juni 2005 (eds D. Gronenborn & J. Petrasch) 175–187 (2010).

[45] Krauß R (2014). Beginnings of the Neolithic in Southeast Europe. The Early Neolithic sequence and absolute dates from Džuljunica-Smărdeš (Bulgaria). Documenta Praehistorica.

[46] Whittle A, Bartosiewicz L, Borić D, Pettit PB, Richards MP (2002). In the beginning: new radiocarbon dates for the Early Neolithic in northern Serbia and south-east Hungary. Antaeus.

[47] Nielsen A, Steinheim G, Mysterud A (2013). Do different sheep breeds show equal responses to climate fluctuations?. Basic and Applied Ecology.

[48] Atti N, Bocquier F, Khaldi G (2004). Performance of the fat-tailed barbarine sheep in its environment: adaptive capacity to alternation of underfeeding and re-feeding periods. Anim. Res..

[49] Dwyer CM (2009). Welfare of sheep: providing for welfare in an extensive environment. Small Rum. Res..

[50] Nielsen A (2012). Are responses of herbivores to environmental variability spatially consistent in alpine ecosystems?. Global Change Biology.

[51] Balasse M, Tresset A (2007). Environmental constraints on reproductive activity of domestic sheep and cattle: what latitude for the herder?. Anthropozoologica.

[52] Balasse, M. *et al*. Sheep birth distribution in past herds: a review for prehistoric Europe (6th – 3rd millennia BC). *Animal: An International Journal of Animal Bioscience* (2017). 10.1017/S1751731117001045 28532521

[53] Özbal H (2013). Neolitik Batı Anadolu ve Marmara Yerleşimleri Çanak Çömleklerinde Organik Kalıntı Analizleri. Arkeometri Sonucları Toplantısı.

[54] Ingold, T. Hunters, pastoralists and ranchers: reindeer economies and their transformations (Cambridge Univ. Press, 1980).

[55] Šoberl L, Gašparič AZ, Budja M, Evershed RP (2008). Early herding practices revealed through organic residue analysis of pottery from the early Neolithic rock shelter of Mala Triglavca, Slovenia. Documenta Praehistorica.

[56] Šoberl L (2014). Neolithic and Eneolithic activities inferred from organic residue analysis of pottery from Mala Triglavca, Moverna vas and Ajdovska jama, Slovenia. Documenta Praehistorica.

[57] Burger J, Kirchner M, Bramanti B, Haak W, Thomas MG (2007). Absence of the lactase–persistence–associated allele in Early Neolithic Europeans. PNAS.

[58] Malmström H (2010). High frequency of lactose intolerance in a prehistoric hunter-gatherer population in northern Europe. BMC Evol Biol..

[59] Lacan M (2011). Ancient DNA reveals male diffusion through the Neolithic Mediterranean route. PNAS.

[60] Lacan M (2011). Ancient DNA suggests the leading role played by men in the Neolithic dissemination. PNAS.

[61] Sverrisdóttir OO (2014). Direct estimates of natural selection in Iberia indicate calcium absorption was not the only driver of lactase persistence in Europe. Mol. Biol. Evol..

[62] Oross K (2016). The early days of Neolithic Alsónyék: the Starčevo occupation. Berichte der Römisch-Germanischen Kommission.

[63] Roodenberg, J., Leshtakov, K. & Petrova, V. *Yabalkovo, Volume 1* (Sofia University, 2014).

[64] Whittle, A. The Early Neolithic on the Great Hungarian Plain. Investigations of the Körös Culture site of Ecsegfalva 23, County Békés. Varia Archaeologica Hungarica. (2007).

[65] Vuković, J. In Beginnings - new research in the appearance of the Neolithic between Northwest Anatolia and the Carpathian Basin (eds R. Krauß) 205–212 (Verlag Marie Leidorf, 2011).

[66] McPherron, A. & Srejović, D. Divostin and the Neolithic of Central Serbia (1988).

[67] Bogdanović, M. Grivac. Naselja Protostarčevačke i Vinčanske kulture (2004).

[68] Correa-Ascencio M, Evershed RP (2014). High throughput screening of organic residues in archaeological potsherds using direct methanolic acid extraction. Analytical Methods.

[69] Greenfield H, Arnold E (2015). ‘Go(a)t milk?’ New perspectives on the zooarchaeological evidence for the earliest intensification of dairying in south eastern Europe. World Archaeology.

[70] Halstead P, Jones G (1980). Early Neolithic economy in Thessaly: some evidence from excavations at Prodromos. Anthropologika (Athen).

[71] El Susi, G. In The First Neolithic Sites in Central/South- East European Transect Volume II: Early Neolithic (Starčevo-Criş) sites on the territory of Romania. British Archaeological Reports International Series 218 (eds S.A. Luca & C. Suciu) 47–56 (Archaeopress, 2011).

[72] Clason, A. T. In *Domestikationsforschung und Geschichte der Haustiere: Internationales Symposion in* Budapest*, 1971* (ed J. Matolcsi) 205–212 (Akadémiai Kiadó, 1973).

[73] Russell, N. & Martin, L. In *Inhabiting Çatalhöyük: Reports from the 1995-1999 Seasons* McDonald *Institute Monographs* (eds I. Hodder) 33–98 (McDonald Institute for Archaeological Research, 2005).

