# Supplementary material for: Earliest expansion of animal husbandry beyond the Mediterranean zone in the sixth millennium BC
Source: Sci Rep. 2017 Aug 2;7:7146. doi: 10.1038/s41598-017-07427-x (PMC5541088; doi:10.1038/s41598-017-07427-x)
Supplement: Supplementary file 1 — Supplementary Information [file 41598_2017_7427_MOESM1_ESM.doc]

**Earliest expansion of animal husbandry beyond the Mediterranean zone in the sixth millennium BC**

**Jonathan Ethier, Eszter Bánffy, Jasna Vuković, Krassimir Leshtakov, Krum Bacvarov, Mélanie Roffet-Salque, Richard P. Evershed, Maria Ivanova^*^**

Supplementary information

Description of sites and stratigraphic contexts of pottery samples

**Bulgaria**

Yabalkovo

Yabalkovo is located in the valley of Maritsa, southeast Bulgaria. The investigations at the site were part of a rescue operation due to infrastructure projects. In the years 2000-2012 more than 25 000 square meters were under exploration. In contrast to the typical tell sites from Upper Thrace, Yabalkovo is described as a “flat” settlement with a vast inhabited area which, according to the scatter of archaeological materials, was estimated to cover c. 1.5 km x 2 km. The excavations revealed several phases of occupation spanning a very long period from the early 6^th^ millennium BC to the 11^th^ century-first quarter of the 13^th^ century CE (the Early and Middle Neolithic, Early Chalcolithic, Early Bronze Age, Late Iron Age, Late Roman Age, and the Middle Ages). The geophysical survey and the excavations of the Early Neolithic sites revealed two separate settled areas, each surrounded by a series of concentric ditches. There is stratigraphic evidence for three building phases.These Early Neolithic settlements belong to the Karanovo I group and the ^14^C dates indicate a life span along the first three centuries of the 6^th^ millennium BC (Table S3). Samples were selected from the excavation campaigns in 2010, 2011 and 2012 and originated from two different areas: 1) Ditches A, A1, House 1 and a Pit 1 in Sector Southwest, and 2) a concentration of finds, part of a large area of rubbish deposition, in Square N35 in Sector North.

Nova Nadezhda

The site is situated on a low-lying terrace in the fertile floodplain of the Middle Maritsa Valley, c. 300 m from the river’s right bank, and covers an area of over 5 ha. It

consists of two low mounds, separated by a stream. In 2013-2014 rescue excavation for a railway construction took place on the north edge of the northwest mound in an area of 7500 m^2^. This part of the site was continually occupied from the beginning of the Early Neolithic (c. 6000 calBC) to the Final Chalcolithic (c. 4000 calBC), and then again in the Early Iron Age (11th–9th c. calBC). According to the radiocarbon dates, the Early Neolithic occupation started at c. 6000 BC and had a duration of c. 400 years (Table S3). Major archaeological features from this phase include five concentric ditches and at least one enclosure wall of wooden posts, as well as the remains of one burnt house^1,2^. The pottery samples are from the 2013 campaign and originate from the house (001) and from various locations in the fills of four enclosure ditches (003, 053, 054, 055).

**Serbia**

Blagotin

Blagotin is situated in the northern outskirt of the village of Poljna near the foothills of the Blagotin Mountain. The site was discovered in 1984 during a survey of the region. Systematic excavations were conducted between 1989 and 2000 on a total area of c. 300 m^2^. Blagotin was a small settlement with a surface area of 0.5 ha, which provided important information about settlement life and architecture during the Early Neolithic, including seven semi-subterranean dwellings and numerous areas of rubbish deposition and pits. The ^14^C dates from this period of habitation have values around 6200-6000 calBC (Table S3). The site was reoccupied twice, during the Eneolithic (Baden-Kostolac culture) and the Early Iron Age (Hallstatt culture)^3-6^. All samples were selected from the 1995 excavations and originate from different stratigraphic levels and locations in the fill of Structure 3, a semi-subterranean dwelling.

Divostin

Discovered in 1952 during a survey led by the Archaeological Institute in Belgrade, the site of Divostin is considered as one of the major settlements of the Starčevo culture. The excavations took place in 1967 (by M. Bogdanovic) and between 1968-1974 (by D. Srejovic and A. McPherron) on a total area of c. 2,400 m^2^. Divostin was first inhabited by Starčevo groups (Divostin I). Five radiocarbon dates on wood charcoal from this period of habitation have values of c. 6000-5700 calBC (Table S3). From the Divostin I phase, semi-subterranean huts and surface-level houses, fireplaces, hearths and ovens, rubbish pits and refuse areas were uncovered. Following a gap of a thousand years, the site was resettled by Late Vinča groups (Divostin II)^7^. The samples belonged to a pottery assemblage derived from the 1967 excavation of M. Bogdanovic of a pit of the Divostin I period in Sector D.

Grivac

The site is located in the locality of Barice near the modern village of Grivac. Habitation remains from the Early Neolithic (Grivac I-III) and Late Neolithic (Grivac IV-VI) were recorded at this site on a total area of c. 25 ha. Series of trenches were excavated in different parts of the site in 1953-1954, as well as in 1957, 1969, and 1989-1994. Features from the Early Neolithic phase included semi-subterranean and above-ground houses^8^. There is one radiocarbon date from this phase, from Trench B, with a value of c. 6200-6000 calBC (Table S3). The pottery samples originate from two separate areas: 1) from the west part of the site (Trench IV and V from 1957, Trench A and B from 1969 and Trench A from 1990); 2) from the east part (Trench 1954 and Trench A from 1994).

**Hungary**

Alsónyék

The site of Alsónyék is located near the Danube in the southern part of the Sárköz region in the Tolna County, at the border of the Transdanubian Hills and the Great Hungarian Plain. The investigations at Alsónyék were conducted between 2006 and 2009 as a part of the rescue excavations along the M6 motorway on an area of c. 25 ha. The site was occupied during the Neolithic and Early Chalcolithic periods (Starčevo, LBK, Sopot and Lengyel culture groups). Alsónyék was the first large-scale excavation at a Starčevo site in Hungary and provided the largest Starčevo assemblage in this region so far. Occupation took place c. 5800-5730 cal BC (Table S3). The excavations identified two separate foci of Starčevo habitation, 10B and 5603. In the larger area 5603 some 500 features, mostly pits of various sizes and shapes, were assigned to the Starčevo period. Other Early Neolithic features included numerous well-preserved ovens, wells, and piles of burnt daub^9,10^. The samples originate from the fills of eleven pits in Area 5603, the main area of Starčevo occupation at Alsónyék.

Ecsegfalva 23

The site of Ecsegfalva was discovered in 1998 during large-scale archaeological surveys along the Kiri-Tó river. The excavations took place from 1999 to 2001 near an old meander of the Hortobágy-Berettyó river, northern tributary of Körös. Small-scale intensive excavations focused on three locations: Ecsegfalva 16, 18, and 23. A total of three trenches was opened at Ecsegfalva 23. The occupation of this location lasted for a short period of time with a starting date around 5800 calBC and peak activity between 5750 and 5650 cal BC (Table S3). Samples from the excavation campaigns in 1999-2001 were selected from all three trenches at Ecsegfalva 23: Area 23A (2 samples), Area 23B (31 samples) and 23C (8 samples).

Supplementary Table S1 Description of samples containing significant concentrations of archaeological lipid residues (FA, fatty acids).

| **Site** | **Lab number** | **Vessel part** | **Context type and location** | **Lipids classes** | **δ^13^C_16:0_ (‰)** | **δ^13^C_18:0_ (‰)** | **∆^13^C (‰)** | **Lipid concen-tration (**µg g^-1^) | **Predominant commodity** |
| --- | --- | --- | --- | --- | --- | --- | --- | --- | --- |
|
| Yabalkovo | YAB 05 | Rim | Pit fill, Pit 1 | FA | -27.2 | -27.1 | 0.1 | 82 | Non-Ruminant Adipose Fats |
|  | YAB 07 | Body | Ditch fill, A1/10 | FA | -27.3 | -26.0 | 1.3 | 1356 | Non-Ruminant Adipose Fats |
|  | YAB 11 | Body | Ditch fill, A/01 | FA | -27.4 | -29.0 | -1.6 | 89 | Ruminant Adipose Fats |
|  | YAB 22 | Rim | Concentration of finds, N35 | FA | -28.0 | -32.6 | -4.6 | 54 | Ruminant Dairy Fats |
|  | YAB 24 | Base | Concentration of finds, N35 | FA | -28.8 | -29.0 | -0.2 | 49 | Non-Ruminant Adipose Fats |
|  | YAB 27 | Base | Concentration of finds, N35 | FA | -26.8 | -31.8 | -5.1 | 545 | Ruminant Dairy Fats |
|  | YAB 30 | Rim | Concentration of finds, N35 | FA | -25.1 | -27.0 | -2.0 | 151 | Ruminant Adipose Fats |
|  | YAB 31 | Rim | Concentration of finds, N35 | FA | -27.7 | -31.4 | -3.7 | 765 | Ruminant Dairy Fats |
|  | YAB 32 | Body | Concentration of finds, N35 | FA | -26.8 | -28.1 | -1.3 | 2632 | Ruminant Adipose Fats |
|  | YAB 33 | Body | Concentration of finds, N35 | FA | -26.1 | -28.9 | -2.8 | 3843 | Ruminant Adipose Fats |
|  | YAB 35 | Rim | Concentration of finds, N35 | FA | -24.8 | -27.6 | -2.8 | 266 | Ruminant Adipose Fats |
|  | YAB 37 | Base | Concentration of finds, N35 | FA | -27.6 | -28.7 | -1.1 | 5956 | Ruminant Adipose Fats |
|  | YAB 38 | Body | Concentration of finds, N35 | FA | -25.8 | -29.7 | -3.9 | 768 | Ruminant Dairy Fats |
|  | YAB 39 | Base | Concentration of finds, N35 | FA | -27.3 | -30.3 | -2.9 | 1169 | Ruminant Adipose Fats |
|  | YAB 40 | Base | Concentration of finds, N35 | FA | -28.0 | -29.6 | -1.7 | 726 | Ruminant Adipose Fats |
| Nova Nadezhda | NNA 02 | Rim | House, 001 | FA | -28.8 | -31.5 | -2.8 | 53 | Ruminant Adipose Fats |
|  | NNA 07 | Rim | House, 001 | FA | -26.4 | -30.8 | -4.3 | 453 | Ruminant Adipose Fats |
|  | NNA 13 | Body | Ditch fill, 003 | FA | -27.7 | -28.6 | -0.9 | 146 | Ruminant Adipose Fats |
|  | NNA 20 | Rim | Ditch fill, 054/2 | FA | -28.2 | -30.0 | -1.8 | 138 | Ruminant Adipose Fats |
|  | NNA 21 | Rim | Ditch fill, 054/2 | FA | -25.7 | -24.6 | 1.1 | 63 | Non-Ruminant Adipose Fats |
|  | NNA 23 | Base | Ditch fill, 054/3 | FA | -28.4 | -29.9 | -1.5 | 54 | Ruminant Adipose Fats |
|  | NNA 32 | Rim | Ditch fill, 003 | FA | -28.1 | -31.0 | -2.8 | 353 | Ruminant Adipose Fats |
|  | NNA 33 | Rim | Ditch fill, 003 | FA | -25.8 | -29.4 | -3.6 | 146 | Ruminant Adipose Fats |
|  | NNA 38 | Rim | Ditch fill, 055 | FA | -27.4 | -28.7 | -1.3 | 254 | Ruminant Adipose Fats |
| Blagotin | BLA 17 | Body | Pit fill, Ql/12 | FA | -27.4 | -31.8 | -4.5 | 463 | Ruminant Dairy Fats |
|  | BLA 20 | Rim | Pit fill, Q/18 | FA | -24.7 | -29.4 | -4.6 | 572 | Ruminant Dairy Fats |
|  | BLA 23 | Rim | Pit fill, Qg/09 | FA | -28.4 | -31.6 | -3.2 | 5679 | Ruminant Adipose Fats |
|  | BLA 28 | Body | Pit fill, Qi/12 | FA | -28.9 | -34.2 | -5.3 | 783 | Ruminant Dairy Fats |
|  | BLA 29 | Rim | Pit fill, Qd/11 | FA | -28.0 | -32.2 | -4.2 | 235 | Ruminant Dairy Fats |
|  | BLA 30 | Rim | Pit fill, Rh/14 | FA | -29.3 | -31.9 | -2.6 | 752 | Ruminant Adipose Fats |
|  | BLA 32 | Rim | Pit fill, Rh/14 | FA | -29.9 | -34.6 | -4.7 | 1937 | Ruminant Dairy Fats |
|  | BLA 34 | Rim | Pit fill, Ql/11 | FA | -27.3 | -31.9 | -4.6 | 93 | Ruminant Dairy Fats |
|  | BLA 36 | Body | Pit fill, Ql/09 | FA | -26.5 | -30.6 | -4.0 | 409 | Ruminant Dairy Fats |
| Divostin | DIV 13 | Rim | Pit fill, D-2/V-67 | FA | -30.6 | -29.4 | 1.2 | 102 | Non-Ruminant Adipose Fats |
|  | DIV 21 | Rim | Pit fill, D_1_-2/V-67 | FA | -31.4 | -30.3 | 1.2 | 264 | Non-Ruminant Adipose Fats |
|  | DIV 40 | Rim | Pit fill, D-1/V-67 | FA | -29.1 | -32.7 | -3.6 | 367 | Mixture Ruminant Adipose/Ruminant Dairy Fats |
| Grivac | GRI 03 | Rim | 1969/A/VI | FA | -26.5 | -31.1 | -4.6 | 1673 | Ruminant Dairy Fats |
|  | GRI 06 | Rim | 1969/B/VI | FA | -27.3 | -30.6 | -3.3 | 232 | Mixture Ruminant Adipose/Ruminant Dairy Fats |
|  | GRI 17 | Rim | 1969/B/IV | FA | -28.6 | -31.5 | -2.9 | 1257 | Ruminant Adipose Fats |
|  | GRI 18 | Rim | An 10/41 | FA | -26.1 | -27.9 | -1.8 | 541 | Ruminant Adipose Fats |
|  | GRI 26 | Rim | 1969/A/V, 14 | FA | -27.6 | -30.2 | -2.6 | 233 | Ruminant Adipose Fats |
|  | GRI 32 | Rim | 1994/A/VIIc | FA | -28.5 | -32.1 | -4.0 | 219 | Ruminant Dairy Fats |
| Alsonyek | ALS 01 | Rim | Pit fill, 704 | FA | -27.1 | -5.1 | -5.1 | 1352 | Ruminant Dairy Fats |
|  | ALS 10 | Rim | Pit fill, 617 | FA | -26.1 | -32.2 | -4.6 | 503 | Ruminant Dairy Fats |
|  | ALS 11 | Rim | Pit fill, 617 | FA | -27.9 | -31.8 | -4.0 | 624 | Ruminant Dairy Fats |
|  | ALS 12 | Rim | Pit fill, 617 | FA | -27.7 | -31.9 | -4.2 | 881 | Ruminant Dairy Fats |
|  | ALS 18 | Rim | Pit fill, 720 | FA | -28.9 | -33.4 | -4.5 | 903 | Ruminant Dairy Fats |
|  | ALS 22 | Rim | Pit fill, 720 | FA | -28.5 | -30.5 | -2.1 | 2265 | Ruminant Adipose Fats |
|  | ALS 23 | Body | Pit fill, 720 | FA | -28.1 | -33.1 | -5.0 | 1398 | Ruminant Dairy Fats |
|  | ALS 24 | Rim | Pit fill, 720 | FA | -28.7 | -33.4 | -4.7 | 774 | Ruminant Dairy Fats |
|  | ALS 25 | Base | Pit fill, 687 | FA | -27.9 | -30.1 | -2.2 | 548 | Ruminant Adipose Fats |
|  | ALS 30 | Rim | Pit fill, 687 | FA | -28.5 | -32.1 | -3.7 | 259 | Ruminant Dairy Fats |
|  | ALS 31 | Rim | Pit fill, 687 | FA | -27.3 | -30.0 | -2.6 | 487 | Ruminant Adipose Fats |
|  | ALS 34 | Rim | Pit fill, 708 | FA | -27.8 | -32.7 | -4.9 | 672 | Ruminant Dairy Fats |
|  | ALS 36 | Rim | Pit fill, 1078 | FA | -27.3 | -29.0 | -1.7 | 152 | Ruminant Adipose Fats |
|  | ALS 38 | Rim | Pit fill, 1078 | FA | -28.3 | -30.9 | -2.6 | 1393 | Ruminant Adipose Fats |
| Ecsegfalva 23 | ECS 08 | Rim | 23C/515 | FA | -26.0 | -30.2 | -4.2 | 177 | Ruminant Dairy Fats |
|  | ECS 16 | Rim | 23B/475 | FA | -27.3 | -31.2 | -3.9 | 912 | Ruminant Dairy Fats |
|  | ESC 17 | Rim | 23B/376 | FA | -28.0 | -25.7 | 2.3 | 1298 | Non-Ruminant Adipose Fats |
|  | ESC 19 | Rim | 23B/376 | FA | -28.1 | -31.0 | -2.9 | 119 | Ruminant Adipose Fats |
|  | ESC 21 | Rim | 23B/430 | FA | -27.4 | -31.9 | -4.5 | 347 | Ruminant Dairy Fats |
|  | ESC 26 | Rim | 23B/464 | FA | -27.5 | -30.0 | -2.5 | 113 | Ruminant Adipose Fats |
|  | ESC 28 | Rim | 23B/301 | FA | -27.6 | -30.9 | -3.2 | 75 | Ruminant Dairy Fats |
|  | ESC 32 | Rim | 23B/301 | FA | -26.9 | -31.0 | -4.0 | 151 | Ruminant Dairy Fats |

Supplementary Table S2. Data on taxonomic abundances in faunal assemblages from early farming sites in the Aegean, the Balkans and the Carpathian Basin.

| **Sites** | **Period/**  **Culture group** | **NISP**** | **Cattle**  **NISP** | **Cattle %** | **Goat**  **NISP** | **Sheep**  **NISP** | **Sheep/Goat**  **NISP** | **Capri-nes %** | **Pig**  **NISP** | **Pig %** | **Large game**  **NISP** | **Large game %** | **Reference** |
| --- | --- | --- | --- | --- | --- | --- | --- | --- | --- | --- | --- | --- | --- |
| **Carpathian Basin** |  | | | | | | | | | | | | |
| Alsónyék | Starčevo | 428 | 124 | 28.97 |  |  | 81 | 18.92 | 17 | 3.97 | 188 | 44 | 11 |
| Ecsegfalva 23 | Körös | 4337 | 436 | 10.05 | 9 | 408 | 3067 | 80.33 | 66 | 1.52 | 248 | 5.82 | 12 |
| Endröd 119 | Körös | 22366 | 5139 | 22.98 | 298 | 2332 | 12717 | 68.62 | 140 | 0.63 | 1645 | 7.35 | 13 |
| Foeni-Gaz | Criș II | 7561 | 2600 | 34.39 |  |  | 3054 | 40.39 | 386 | 5.11 | 1489 | 19.7 | 14 |
| Foeni-Salaș | Criș II | 2563 | 895 | 34.92 | 77 | 270 | 668 | 39.60 | 99 | 3.86 | 536 | 21 | 15 |
| Lánycsók | Starčevo | 1068 | 209 | 19.57 | 21 | 103 | 791 | 85.67 | 16 | 1.50 | 51 | 4.77 | 16 |
| Ludas Budžak | Körös | 2450 | 284 | 11.59 |  |  | 1863 | 76.04 | 8 | 0.33 | 572 | 23.34 | 17 |
| Miercurea Sibiului-Petris | Criș I-III | 890 | 486 | 54.61 |  |  | 262 | 29.44 | 9 | 1.01 | 128 | 16 | 18 |
| Nosa | Körös | 911 | 95 | 10.42 |  |  | 115 | 12.62 | 18 | 1.97 | 736 | 80.79 | 19 |
| Röszke | Körös | 1397 | 153 | 10.95 |  |  | 631 | 45.17 | 14 | 1.00 | 525 | 38.69 | 20 |
| Şeusa-Cărarea Morii | Criș IB-IIA | 1086 | 335 | 30.85 |  |  | 620 | 57.09 | 77 | 7.09 | 51 | 4.7 | 14 |
| Szajol | Körös | 1361 | 576 | 42.32 |  |  | 680 | 49.96 | 2 | 0.15 | 87 | 6.45 | 20 |
| Szolnok-Szanda | Körös | 6556 | 1557 | 23.75 | 58 | 571 | 3428 | 61.88 | 93 | 1.42 | 714 | 11.06 | 20 |
| Tiszaszőlős | Körös | 949 | 242 | 25.50 |  | 137 |  | 14.43 | 33 | 3.47 | 459 | 48.36 | 21 |
| **Northern Balkans** |  | | | | | | | | | | | | |
| Blagotin | Proto- Starčevo | 8706 | 2684 | 30.83 | 281 | 1048 | 3841 | 59.38 | 115 | 1.32 | 722 | 8.29 | 3 |
| Divostin | Proto- Starčevo | 2398 | 1117 | 46.58 | 14 | 92 | 875 | 40.9 | 84 | 3.50 | 200 | 8.34 | 22 |
| Donja Branjevina | Proto- Starčevo | 1926 | 404 | 11.20 |  |  | 1306 | 36.22 | 26 | 0.72 | 1856 | 33.63 | 23 |
| Golokut | Starčevo | 1160 | 260 | 22.41 | 2 | 6 | 118 | 10.86 | 23 | 1.98 | 747 | 64.39 | 24 |
| Koprivec | Criș | 2005 | 976 | 48.68 | 18 | 127 | 645 | 39.40 | 5 | 0.25 | 223 | 11.62 | 25 |
| Lepenski Vir | Proto- Starčevo | 1959 | 375 | 19.14 |  |  | 81 | 4.13 | 8 | 0.4 | 1355 | 69.16 | 26 |
| Magura-Boldullui Moş Ivan̆us* | Criș I | 8500 |  | 31 |  |  |  | 60 |  | 0.1 |  | 8 | 27 |
| Mihajlovac | Starčevo | 2554 | 853 | 33.40 | 35 | 137 | 1268 | 56.38 | 5 | 0.20 | 255 | 9.98 | 28 |
| Ovcharovo-Gorata | Karanovo II | 1251 | 803 | 64.19 | 8 | 6 | 213 | 18.15 | 101 | 8.07 | 114 | 9.11 | 29 |
| Samovodene* | Karanovo II | 2170 |  | 80 |  |  |  |  |  |  |  | 35 | 30 |
| Schela Cladovei | Criș | 1569 | 400 | 25.49 |  | 35 | 292 | 20.84 | 66 | 4.20 | 747 | 47.6 | 31 |
| Starčevo-Grad | Starčevo | 1159 | 505 | 43.57 |  | 4 | 213 | 18.72 | 31 | 2.67 | 403 | 34.77 | 32 |
| **Southern Balkans** |  | | | | | | | | | | | | |
| Anzabegovo I-III | Anzabegovo-Vršnik I-III | 3192 | 305 | 9.56 |  |  | 2476 | 77.57 | 288 | 9.02 | 80 | 2.5 | 33 |
| Kapitan Dimitrievo | Karanovo I | 1650 | 512 | 31.03 |  |  | 932 | 56.48 | 127 | 7.70 | 72 | 4.4 | 34 |
| Karanovo I | Karanovo I | 4671 | 990 | 21 | 76 | 265 | 2234 | 55 | 374 | 8 | 630 | 13,48 | 35 |
| Kovacevo* | Karanovo I |  |  | 14 |  |  |  | 65 |  | 21 |  | 3.5 | 30 |
| Madžari | Anzabegovo-Vršnik IV | 2856 | 1104 | 38.65 | 33 | 145 | 1116 | 45.30 | 273 | 9.55 | 167 | 5.65 | 36 |
| Mursalevo | Karanovo I | 2954 | 364 | 12.32 | 39 | 210 | 1452 | 57.58 | 488 | 16.51 | 394 | 13.33 | 37 |
| Na Breg* | Anzabegovo-Vršnik | 1407 |  | 14.6 |  |  | 76.8 |  |  | 5.7 |  | 2.4 | 38 |
| Rakitovo | Karanovo I | 4373 | 1944 | 44.45 |  |  | 897 | 20.51 | 214 | 4.89 | 1278 | 29.21 | 39 |
| Slatina | Karanovo I | 3861 | 1637 | 42.40 | 33 | 202 | 1268 | 32.84 | 350 | 9.07 | 362 | 9.17 | 40 |
| Yabalkovo | Karanovo I | 2086 | 513 | 24.59 | 12 | 31 | 1298 | 64.29 | 159 | 7.62 | 49 | 2.35 | 41 |
| **Aegean** |  | | | | | | | | | | | | |
| Achilleion II | Ceramic EN | 1489 | 61 | 4.09 |  |  | 1157 | 77.7 | 152 | 10.2 | 73 | 4.9 | 42 |
| Argissa | Aceramic EN | 2195 | 103 | 4.69 |  | 33 | 1820 | 84.42 | 216 | 9.84 | 52 | 2.37 | 43 |
| Franchthi* | Ceramic EN |  |  |  |  |  | 70 |  |  | 30 |  |  | 44 |
| Nea Nikomedia | Ceramic EN | 450 | 64 | 14.44 |  |  | 310 | 68.89 | 65 | 14.44 | 11 | 2,44 | 45 |
| Prodromos 1-2 | Ceramic EN | 1299 | 388 | 29.86 |  |  | 718 | 55.27 | 171 | 13.16 | 22 | 1.69 | 46 |
| Revenia-Korinou | Ceramic EN | 2140 | 257 | 12 |  |  | 1470 | 68.69 | 363 | 16.96 | 30 | 1.40 | 47 |
| Servia* | MN |  |  | 15 |  |  |  | 60 |  | 15 |  | 10 | 48 |
| Sesklo | Ceramic EN | 721 | 92 | 13.80 |  |  | 427 | 64.1 | 144 | 21.6 | 21 | 2.9 | 49 |
| Ulucak V/early | Ceramic EN | 2149 | 394 | 18.33 | 55 | 96 | 1215 | 63.65 | 289 | 13.44 | 63 | 2.9 | 50 |

Dating of periods/culture gorups^51-55^: Proto- Starčevo, Criș I - 6100-5900 BC, Starčevo, Körös, Criș II-III - 5900-5600 BC, Karanovo I, Anzabegovo- Vršnik I-III 6100/6000-5700 BC, Karanovo II, Anzabegovo- Vršnik IV - 5700-5500 BC, Aceramic EN – early 7^th^ mill. BC, Ceramic EN - 6500-5900 BC, MN - 5900-5600 BC

* site not included in Fig. 3

** excludes birds, reptiles, small rodents, fish and molluscs

Supplementary Table S3. Radiocarbon dates from sites sampled for organic residue analysis

| **Site** | **Lab. no.** | **Date BP** | **Material** | **Context** | **References** |
| --- | --- | --- | --- | --- | --- |
| Yabalkovo | OxA-20173 | 7040 ±32 | Human bone | Burial 2 | 56 |
| Yabalkovo | OxA-20174 | 6971 ±35 | Human bone | Burial 1 | 56 |
| Yabalkovo | OxA-23558 | 6930 ±45 | Grain | Pit 1 in AA22–23, 0.4 m | 56 |
| Yabalkovo | OxA-23560 | 6892 ±40 | Grain | Pit 1 in AA24–26, 0.7 m | 56 |
| Yabalkovo | OxA-23561 | 6849 ±39 | Grain | Pit 1 in AA19, 0.5 m | 56 |
| Yabalkovo | OxA-23559 | 6791 ±40 | Grain | Pit 1 in AA22–23, 0.4 m | 56 |
| Yabalkovo | OxA-24530 | 6763 ±36 | Charcoal | Ditch B1, Area 5, 1.50 m | 56 |
| N. Nadezhda | SUERC-53068 | 6979 ± 33 | Large mammal, long bone | Ditch 054/1 | 1 |
| N. Nadezhda | SUERC-53069 | 6895 ± 33 | Large mammal | Ditch 054/3 | 1 |
| N. Nadezhda | SUERC-58062 | 6836 ± 35 | Cattle calcaneus | Ditch 054/2 | 1 |
| N. Nadezhda | SUERC-53067 | 6789 ± 33 | Roe deer femur femurfemurfemur:Capreocapreolus | Ditch 054/1 | 1 |
| N. Nadezhda | SUERC-53070 | 6767 ± 33 | Cattle scapula | Ditch 054/3 | 1 |
| N. Nadezhda | SUERC-58064 | 6749 ± 33 | Cattle vertebrum | Ditch 054/2 | 1 |
| N. Nadezhda | SUERC-53071 | 6746 ± 30 | Sheep tibia | Ditch 054/2 | 1 |
| N. Nadezhda | SUERC-62352 | 6798 ± 41 | Human right tibia | Burial 166 | 1 |
| N. Nadezhda | SUERC-62346 | 6761 ± 41 | Human right tibia | Burial 062 | 1 |
| N. Nadezhda | SUERC-62347 | 6733 ± 41 | Human tibia | Burial 070 | 1 |
| N. Nadezhda | SUERC-62349 | 6712 ± 41 | Human tibia | Burial 085 | 1 |
| N. Nadezhda | SUERC-62342 | 6657 ± 38 | Human left femur | Burial 042 | 1 |
| Blagotin | OxA-8608 | 7480 ±55 | Red deer antler | Pit dwelling ZM7 | 51 |
| Blagotin | OxA-8609 | 7270 ±50 | Human infant bone | Pit dwelling ZM7 | 51 |
| Blagotin | OxA-8760 | 7230 ±50 | Bone perforator | Pit dwelling ZM7 | 51 |
| Divostin | Bln-823 | 7080 ±180 | Charcoal | Feature 15 (earth-cabin 5) | 7 |
| Divostin | Bln-866 | 7060 ±100 | Charcoal | Beneath floor House 17 (Divostin II) | 7 |
| Divostin | Bln-866a | 7200 ±100 | Charcoal | Beneath floor House 17 (Divostin II) | 7 |
| Divostin | Bln-931 | 7050 ±100 | Charcoal | Beneath floor House 17 (Divostin II) | 7 |
| Divostin | Bln-862 | 6995 ±100 | Charcoal | Posthole | 7 |
| Divostin | Bln-899 | 7200 ±100 | Charcoal | Posthole | 7 |
| Divostin | Bln-824 | 6970 ±100 | Charcoal | Feature 15 (earth-cabin 5) | 7 |
| Divostin | Bln-896 | 6945 ±100 | Charcoal | Feature 120E, pit 22 | 57 |
| Divostin | BM-573 | 6935 ±100 | Charcoal | Feature 120E, pit 22 | 57 |
| Grivac | Bln-869 | 7250 ±100 | Charcoal | Pit, Sonda B | 7 |
| Alsónyék | OxA-30230 | 6639 ±35 | Sheep/goat femur | Site 5603, 605/179, pit | 10 |
| Alsónyék | SUERC-51449 | 6886 ±31 | Wild boar radius | Site 5603, 617/222, pit | 10 |
| Alsónyék | OxA-30481 | 6822 ±36 | Sheep/goat centrotarsal | Site 5603, 675/346, pit | 10 |
| Alsónyék | OxA-30231 | 6647 ±37 | Sheep/gat radius | Site 5603, 676/410, pit | 10 |
| Alsónyék | SUERC-51450 | 6590 ±32 | Cattle vertebra | Site 5603, 687/1248, pit | 10 |
| Alsónyék | MAMS-11926 | 6649 ±29 | Human bone | Site 5603, 688, burial | 10 |
| Alsónyék | SUERC-57541 | 6830 ±35 | Cattle humerus | Site 5603, 704/358, pit | 10 |
| Alsónyék | SUERC-51451 | 6656 ±32 | Aurochs tibia | Site 5603, 708/871, pit | 10 |
| Alsónyék | Poz-67492 | 6480 ±40 | Cattle tibia | Site 5603, 708/872, pit | 10 |
| Alsónyék | SUERC-57540 | 6660 ±34 | Wild boar ulna | Site 5603, 720/453, pit | 10 |
| Alsónyék | OxA-X-2586-27 | 6625 ±40 | Cattle radius | Site 5603, 720/848, pit | 10 |
| Alsónyék | MAMS-11927 | 6852 ±31 | Human bone | Site 5603, 721, burial | 10 |
| Alsónyék | MAMS-11928 | 6677 ±27 | Human bone | Site 5603, 745, burial | 10 |
| Alsónyék | MAMS-11929 | 6571 ±34 | Human bone | Site 5603, 746, burial | 10 |
| Alsónyék | MAMS-11930 | 6672 ±35 | Human bone | Site 5603, 775, burial | 10 |
| Alsónyék | MAMS-11931 | 6657 ±30 | Human bone | Site 5603, 797, burial | 10 |
| Alsónyék | SUERC-57542 | 6644 ±36 | Human bone | Site 5603, 1061, burial | 10 |
| Alsónyék | Poz-67494 | 6750 ± 40 | Sheep/goat radius | Site 5603, 1072/1296, oven | 10 |
| Alsónyék | SUERC-5145 | 6903 ± 35 | Sheep/goat tibia | Site 5603, 1078/5112, pit | 10 |
| Alsónyék | MAMS-11932 | 6661 ± 25 | Human bone | Site 5603, 1372, burial | 10 |
| Alsónyék | SUERC-51453 | 6708 ± 33 | Cattle ulna | Site 5603, 1383/1930 | 10 |
| Alsónyék | OxA-30353 | 6738 ± 33 | Human bone | Site 5603, 1398, child burial | 10 |
| Alsónyék | OxA-30354 | 6679 ± 34 | Human bone | Site 5603, 1398, child burial | 10 |
| Alsónyék | OxA-X-2583-19 | 6906 ± 34 | Wild boar femur | Site 5603, 1428/4865, pit | 10 |
| Alsónyék | MAMS-11933 | 6704 ±34 | Human bone | Site 5603, 1435, burial | 10 |
| Alsónyék | MAMS-11934 | 6800 ±35 | Human bone | Site 5603, 1436, burial | 10 |
| Alsónyék | MAMS-11935 | 6857 ±31 | Human bone | Site 5603, 1483, burial | 10 |
| Alsónyék | SUERC-51454 | 6713 ±33 | Sheep/goat metapodial | Site 5603, 1501/2248, pit | 10 |
| Alsónyék | MAMS-11936 | 6698 ±34 | Human bone | Site 5603, 1525, burial | 10 |
| Alsónyék | SUERC-51458 | 6850 ±33 | Sheep/goat femur | Site 5603, 1526/2717, pit | 10 |
| Alsónyék | MAMS-11937 | 6709 ±34 | Human bone | Site 5603, 1527, burial | 10 |
| Alsónyék | MAMS-11938 | 6617 ±38 | Human bone | Site 5603, 1528, burial | 10 |
| Alsónyék | MAMS-11939 | 6695 ±40 | Human bone | Site 5603, 1532, burial | 10 |
| Alsónyék | MAMS-11940 | 6853 ±38 | Human bone | Site 5603, 1333, burial | 10 |
| Ecsegfalva | OxA-11983 | 6915 ±36 | Sheep/goat | Trench 23A | 58 |
| Ecsegfalva | OxA-11871 | 6930 ±40 | Cereal grains | Trench 23A | 58 |
| Ecsegfalva | OxA-11863 | 6825 ±45 | Cereal grains | Trench 23A | 58 |
| Ecsegfalva | OxA-9334 | 6855 ±50 | Emmer | Trench 23A | 58 |
| Ecsegfalva | OxA-9335 | 6920 ±50 | Barley | Trench 23A | 58 |
| Ecsegfalva | OxA-10678 | 6250 ±45 | Human bone | Trench 23A | 58 |
| Ecsegfalva | OxA-12859 | 6818 ±44 | Cattle | Trench 23B | 58 |
| Ecsegfalva | OxA-11982 | 6806 ±39 | Sheep/goat | Trench 23B | 58 |
| Ecsegfalva | OxA-12855 | 6596 ±42 | Sheep | Trench 23B | 58 |
| Ecsegfalva | OxA-11850 | 6780 ±50 | Sheep/goat | Trench 23B | 58 |
| Ecsegfalva | OxA-10501 | 6885 ±50 | Roe deer | Trench 23B | 58 |
| Ecsegfalva | OxA-10500 | 6900 ±60 | Sheep/goat | Trench 23B | 58 |
| Ecsegfalva | OxA-9333 | 6860 ±45 | Cattle | Trench 23B | 58 |
| Ecsegfalva | OxA-13511 | 6785 ±45 | Sheep | Trench 23B | 58 |
| Ecsegfalva | OxA-12857 | 7944 ±44 | Cattle | Trench 23B | 58 |
| Ecsegfalva | OxA-9332 | 6810 ±45 | Sheep | Trench 23B | 58 |
| Ecsegfalva | OxA-9331 | 6815 ±45 | Sheep | Trench 23B | 58 |
| Ecsegfalva | OxA-12858 | 6782 ±42 | Sheep/goat | Trench 23B | 58 |
| Ecsegfalva | OxA-11845 | 6865 ±40 | Cattle | Trench 23B | 58 |
| Ecsegfalva | OxA-12854 | 6774 ±45 | Sheep/goat | Trench 23B | 58 |
| Ecsegfalva | OxA-X-2040-09 | 6780 ±39 | Sheep/goat | Trench 23B | 58 |
| Ecsegfalva | OxA-9328 | 6815 ±50 | Large mammal | Trench 23B | 58 |
| Ecsegfalva | OxA-X-2040-08 | 6775 ±37 | Sheep/goat | Trench 23B | 58 |
| Ecsegfalva | OxA-X-2040-07 | 6787 ±37 | Sheep/goat | Trench 23B | 58 |
| Ecsegfalva | OxA-9330 | 6795 ±50 | Sheep/goat | Trench 23B | 58 |
| Ecsegfalva | OxA-9325 | 6690 ±50 | Equid | Trench 23B | 58 |
| Ecsegfalva | OxA-10148 | 6665 ±50 | Equid | Trench 23B | 58 |
| Ecsegfalva | OxA-12140 | 6729 ±32 | Sheep | Trench 23B | 58 |
| Ecsegfalva | OxA-13510 | 6731 ±43 | Sheep/goat | Trench 23B | 58 |
| Ecsegfalva | OxA-12860 | 6826 ±41 | Cattle | Trench 23C | 58 |
| Ecsegfalva | OxA-11984 | 6893 ±36 | Sheep | Trench 23C | 58 |
| Ecsegfalva | OxA-10505 | 6845 ±50 | Sheep/goat | Trench 23C | 58 |
| Ecsegfalva | OxA-12141 | 1335 ±26 | Human bone | Trench 23C | 58 |
| Ecsegfalva | OxA-11868 | 6750 ±45 | Cattle | Trench 23C, Pit 393 | 58 |
| Ecsegfalva | OxA-11849 | 6660 ±40 | Animal bone | Trench 23C, Pit 394 | 58 |
| Ecsegfalva | OxA-12655 | 6830 ±35 | Sheep/goat | Trench 23C, Pit 395 | 58 |
| Ecsegfalva | OxA-12654 | 6889 ±36 | Sheep/goat | Trench 23C, Pit 396 | 58 |

References:

1 Bacvarov, K., Todorova, N., Katsarov, G. & Petrova, V. *Lapped by the river Maritsa: rescue excavations at the prehistoric and protohistoric site of Nova Nadezhda, Southeast Bulgaria (forthcoming).* (2017).

2 Bacvarov, K., Todorova, N., Katsarov, G., Petrova, V. & Mcsweeney, K. in *Southeast Europe and Anatolia in prehistory. Essays in honor of Vassil Nikolov on his 65th anniversary* (eds K. Bacvarov & R. Gleser) 149-158 (Rudolf Habelt, 2016).

3 Greenfield, H. J., Greenfield, T. L. J. & Jezik, S. Subsistence and settlement in the Early Neolithic of temperate SE Europe: a view from Blagotin, Serbia. *Archaeologia Bulgarica* **18**, 1-33 (2014).

4 Jezik, S. S. *The Origins of Agriculture in Temperate Europe: An Exploration into the Subsistence Strategies of Two Early Neolithic Groups in the Central Balkans, Foeni-Salas and Blagotin*, (1998).

5 Stankovic, S., Redzic, M. & Zecevic, J. Arheoloska iskopavanja na lokalitetu Blagotin u 1996. *Glasnik SAD* **13**, 95-101 (1997).

6 Vuković, J. in *Beginnings - new research in the appearance of the Neolithic between Northwest Anatolia and the Carpathian Basin* (ed R. Krauß) 205-212 (Verlag Marie Leidorf, 2011).

7 McPherron, A. & Srejović, D. *Divostin and the Neolithic of Central Serbia*. (1988).

8 Bogdanović, M. *Grivac. Naselja Protostarčevačke i Vinčanske kulture*. (2004).

9 Bánffy, E., Marton, T. & Osztás, A. in *Neolithisation of the Carpathian Basin: Northernmost distribution of the Starcevo/Körös culture* (eds J. K. Kozlowski & P. Raczky) 37-51 (2010).

10 Oross, K. *et al.* The early days of Neolithic Alsónyék: the Starčevo occupation. *Berichte der Römisch-Germanischen Kommission* **94**, 93-121 (2016).

11 Nyerges, E. A. Preliminary report on the Neolithic archaeozoological finds from Alsonyek-Bataszek, Hungary. *Archeometriai Műhely* **10**, 209-214 (2013).

12 Bartosiewicz, L. in *The Early Neolithic on the Great Hungarian Plain: investigation of the Körös culture site of Ecsegfalva 23, County Békés* Vol. Varia Archaeologica XXI (ed A. Whittle) 287-326 (Institute of Archaeology of the Hungarian Academy of Sciences, 2007).

13 Bökönyi, S. *Cultural and landscape changes in South-east Hungary I. Reports on the Gyomaendrőd Project*. (Archeolingua, 1992).

14 El Susi, G. The comparative analysis of faunal samples from sites dated in Starčevo-Körös-Criş culture – phases Ib-IIa from Transylvania and Banat. *Acta Terrae Septemcastrensis* **7**, 91-106 (2008).

15 Greenfield, H. J. & Jongsma, T. L. in *Living well together? : settlement and materiality in the Neolithic of South-East and Central Europe* (eds D.W. Bailey, A. Whittle, & D. Hofmann) 108-130 (Oxbow, 2008).

16 Bökönyi, S. Early Neolithic vertebrate fauna from Lanycsok-Égettmalom. *Acta Archaeologica Academiae Scientiarum Hungaricae* **33**, 21-34 (1981).

17 Bökönyi, S. *History of Domestic Mammals in Central and Eastern Europe*. (1974).

18 El Susi, G. in *The First Neolithic Sites in Central/South- East European Transect Volume II: Early Neolithic (Starčevo-Criş) sites on the territory of Romania. British Archaeological Reports International Series 218* (eds S.A. Luca & C. Suciu) 47–56 (Archaeopress, 2011).

19 Bökönyi, S. Die frühneolithische Wirbeltierfauna von Nosa. *Acta Archaeologica Academiae Scientiarum Hungaricae* **36**, 29-41 (1984).

20 Bartosiewicz, L. in *The First Neolithic Sites in Central/ South-East European Transect. Volume III. The Körös Culture in Eastern Hungary. British Archaeological Reports, IBAR International Series 2334* (eds A. Anders & Z. Siklósi) 195-204 (Archaeopress, 2012).

21 Domboroczki, L. in *Neolithization of the Carpathian Basin: Norhternmost distribution of the Starcevo/Körös culture* (eds J. K. Kozlowski & P. Raczky) 137-176 (2010).

22 Bökönyi, S. in *Divostin and the Neolithic of Central Serbia* *Ethnology monograph* (eds Alan McPherron & Dragoslav Srejovic) 419-445 (Dept. of Anthropology, University of Pittsburgh, 1988).

23 Blažić, S. in *Donja Branjevina. A neolithic settlement near Deronje in the Vojvodina (Serbia)* (ed J. Karmanski) 74-76 (2005).

24 Blažić, S. Ostaci faune sa arheološkog nalazišta kod Vizića. *Rad Vojvođanskih Muzeja*, 33–36 (1985).

25 Manhart, H. Die vorgeschichtliche Tierwelt von Koprivec und Durankulak und anderen prähistorischen Fundplätzen in Bulgarien aufgrund von Knochenfunden aus archäologischen Ausgrabungen. *Documenta naturae* **116** (1998).

26 Bökönyi, S. in *Lepenski Vir : nova praistorijska kultur u podunavlu* (ed Dragoslav Srejović) Ch. 328 S., 224-228 (Srpska Kniz. Zadura, 1969).

27 Balasse, M. *et al.* Early herding at Magura-Boldul lui Mos, Ivanus (early sixth millennium BC, Romania): environments and seasonality from stable isotope analysis. *European Journal of Archaeology* **16**, 221-246 (2013).

28 Bökönyi, S. Animal remains of Mihajlovac-Knjepiste. *Balcanica* **23**, 77-87 (1992 ).

29 Nobis, G. Zur Fauna der frühneolithischen Siedlung Ovcarovo-Gorata, Bez. Targoviste (NO-Bulgarien). *Bonner zoologische Beiträge* **37**, 1-22 (1986).

30 Benecke, N. in *Aegean – Marmara – Black Sea: The Present State of Research in the Early Neolithic* (eds I. Gatsov & H. Schwarzberg) 175–185 (Beier and Beran, 2006).

31 Bartosiewicz, L., Boroneant, V., Bonsall, C. & Stallibrass, S. in *From the mesolithic to the neolithic : proceedings of the International Archaeological Conference "From the Mesolithic to the Neolithic", held in the Damjanich Museum of Szolnok, September 22 - 27, 1996* Vol. 11 *Archaeolingua* (eds R. Kertész & J. Makkay) 15-22 (Archaeolingua Alapítvány, 2001).

32 Clason, A. T. Padina and Starcevo: Game, fish and cattle. *Palaeohistoria* **22**, 141-173 (1980).

33 Bökönyi, S. in *Neolithic Macedonia: as reflected by excavations at Anza, Southeast Yugoslavia* (ed Marija Gimbutas) 313-363 (Univ. of California, 1976).

34 Ninov, L. in *Selistna mogila Kapitan Dimitrievo: razkopki 1998-1999* (ed Macanova V. Nikolov V., Stefanova T., Bozhilov V., Bachvarov K., Gacov I, Marinova E. and Ninov L.) Ch. IX, 131-136 (Archeologiceski Institut s Muzej - BAN, 1999).

35 Bökönyi, S. & Bartosiewicz, L. in *Karanovo Bd.1: Die Ausgrabungen im Südsektor 1984-1992* (ed Hiller S. and Institut für Klassische Archäologie) Ch. 480 S., 385-423 (Berger, 1997).

36 Moskalewska, A. L. & Sanev, V. Preliminary Analysis of Bone Remnants of Animals from the Neolithic Archaeological Site Tumba Madžari near Skopje (Yugoslavia). *Macedoniae Acta Archaeologica* **10**, 55–75 (1989).

37 Marinova, E., De Cupere, B. & Nikolov, V. in *Southeast Europe and Anatolia in prehistory. Essays in honor of Vassil Nikolov on his 65th anniversary* (eds R. Gleser & K. Bacvarov) 509-519 (Dr. Rudolf Habelt, 2016).

38 Orton, D. Herding, settlement, and chronology in the Balkan Neolithic. *European Journal of Archaeology* **15**, 5-40 (2012).

39 Kovachev, G. & Georgiev, G. in *Neolitnoto selishte do grad Rakitovo [Neolithic settlement near Rakitovo]* Vol. Razkopki i Proucvanija (eds A. Raduncheva *et al.*) 171-190 (Gal-Iko Publisher, 2002).

40 Bökönyi, S. Eine vorläufige Mitteilung über die Tierknochenfunde von Sofia-Slatina, Bauhorizont l. *Acta Praehistorica et Archaeologica* **24**, 245-247 (1992).

41 Spassov, N. & Iliev, N. in *Yabalkovo* Vol. I (eds Roodenberg J., K. Leshtakov, & V. Petrova) Ch. XII, 425-432 (ATE - Ars et Technica Explicatus, 2014).

42 Bökönyi, S. in *Achilleion: a Neolithic settlement in Thessaly, Greece, 6400 - 5600 BC* Vol. Monumenta Archaeologica (eds M. Gimbutas, S.M.N. Winn, & D.M. Shimabuku) 315-332 (Univ. of California, 1989).

43 Boessneck, J. in *Die deutschen Ausgrabungen auf der Argissa-Magula in Thessalien 1. Das präkeramische Neolithikum sowie die Tier- und Pflanzenreste* (eds V. Milojcic, J. Boessneck, & M. Hopf) 27-99 (Habelt, 1962).

44 Payne, S. in *Archaeozoological studies* (ed A. T. Clason) 120-131 (North-Holland Publisher, 1975).

45 Higgs, E. S. Fauna, in: Rodden, Robert J., Excavations at the early neolithic site at Nea Nikomedeia, Greek Macedonia (1961 season). *Proceedings of the Prehistoric society* **28**, 271-274 (1962).

46 Halstead, P. & Jones, G. Early Neolithic economy in Thessaly: some evidence from excavations at Prodromos. *Anthropologika (Athen)* **1**, 93-117 (1980).

47 Halstead, P. & Isaakidou, V. in *The Origins and Spread of Stock-Keeping in the Near East and Europe* (eds S. Colledge, J. Connoly, K. Dobney, & S. Shennan) 129–144 (Left Coast Press, 2013).

48 Watson, J. P. N. Faunal remains, in Ridley, Cressida, Wardle, K. A. Rescue excavations at Servia 1971-73: a preliminary report. *The Annual of the British School at Athens*, 228-229 (1979).

49 Schwartz, C. A. The fauna from Early Neolithic Sesklo, in Wijnen, M.-H., The early neolithic I settlement at Sesklo : an early farming community in Thessaly, Greece. *Analecta Praehistorica Leidensia* **14**, 134-136 (1982).

50 Çakırlar, C. The evolution of animal husbandry in Neolithic central-west Anatolia: The zooarchaeological record from Ulucak Höyük (c. 7040–5660 cal. BC, Izmir, Turkey). *Anatolian Studies* **62**, 1-33 (2012).

51 Whittle, A., Bartosiewicz, L., Borić, D., Pettit, P. B. & Richards, M. P. In the beginning: new radiocarbon dates for the Early Neolithic in northern Serbia and south-east Hungary. *Antaeus* **25**, 63–117 (2002).

52 Oross, K. & Siklósi, S. in *The first Neolithic sites in central / south-east European transect III. The Körös culture in eastern Hungary (BAR Internat. Ser. 2334)* (eds A. Anders & Zs. Siklósi) 129–159 (Archaeopress, 2012).

53 Reingruber, A. & Thissen, L. Depending on 14C data: Chronological frameworks in the neolithic and chalcolithic of southeastern Europe. *Radiocarbon* **51**, 751-770 (2009).

54 Reingruber, A. & Thissen, L. in *How did farming reach Europe? Anatolian-European relations from the second half of the 7th through the rst half of the 6th millennium cal BC. BYZAS 2* (ed C. Lichter) 295−327 (2005).

55 Krauß, R. in *Panta Rhei: Studies on the Chronology and Cultural Development of South-Eastern and Central Europe in Earlier Prehistory Presented to Juraj Pavúk on the Occasion of his 75th Birthday* (eds J. Šuteková, P. Pavúk, P. Kalábková, & B. Kovár) 35-58 (University of Bratislava and Archaeological Centre Olomouc, 2010).

56 Roodenberg, J., Leshtakov, K. & Petrova, V. *Yabalkovo, Volume 1*. (Sofia University, 2014).

57 Burleigh, R., Hewson, A. & Meeks, N. British Museum natural radiocarbon measurements IX. *Radiocarbon* **19**, 143-160 (1977).

58 Bronk Ramsey, C., Higham, T., Whittle, A. & Bartosiewicz, L. in *The Early Neolithic of the Great Hungarian Plain. Investigations of the Körös culture site of Ecsegfalva 23, County Békés. (= Varia Archaeologica Hungarica 21)* (ed Whittle A.) 173–188 (2007).
